# Transaldolase haploinsufficiency in subjects with acetaminophen‐induced liver failure

**DOI:** 10.1002/jimd.12197

**Published:** 2020-01-01

**Authors:** Zachary Oaks, John Jimah, Craig C. Grossman, Miguel Beckford, Ryan Kelly, Sanjay Banerjee, Brian Niland, Gabriella Miklossy, Zarife Kuloglu, Aydan Kansu, William Lee, Laszlo Szonyi, Katalin Banki, Andras Perl

**Affiliations:** ^1^ Department of Medicine, State University of New York Upstate Medical University Syracuse New York; ^2^ Department of Pediatric Gastroenterology and Hepatology Ankara University School of Medicine Ankara Turkey; ^3^ Department of Medicine University of Texas Southwestern Medical Center Dallas Texas; ^4^ Department of Pediatrics I Semmelweis University Budapest Hungary; ^5^ Department of Pathology, State University of New York Upstate Medical University Syracuse New York

**Keywords:** acetaminophen, liver disease, prevalence, transaldolase deficiency, variations

## Abstract

Transaldolase (TAL) is an enzyme in the pentose phosphate pathway (PPP) that generates NADPH for protection against oxidative stress. While deficiency of other PPP enzymes, such as transketolase (*TKT*), are incompatible with mammalian cell survival, mice lacking TAL are viable and develop progressive liver disease attributed to oxidative stress. Mice with homozygous or heterozygous TAL deficiency are predisposed to cirrhosis, hepatocellular carcinoma (HCC) and acetaminophen (APAP)‐induced liver failure. Both mice and humans with complete TAL deficiency accumulate sedoheptulose 7‐phosphate (S7P). Previous human studies relied on screening patients with S7P accumulation, thus excluding potentially pathogenic haploinsufficiency. Of note, mice with TAL haploinsufficiency are also predisposed to HCC and APAP‐induced liver failure which are preventable with oral N‐acetylcysteine (NAC) administration. Based on *TALDO1* DNA sequencing, we detected functional TAL deficiency due to novel, heterozygous variations in two of 94 healthy adults and four of 27 subjects with APAP‐induced liver failure (*P* = .022). The functional consequences of these variations were individually validated by site‐directed mutagenesis of normal cDNA and loss of activity by recombinant enzyme. All four patients with TAL haplo‐insufficiency with APAP‐induced liver failure were successfully treated with NAC. We also document two novel variations in two of 15 children with previously unexplained liver cirrhosis. Examination of the National Center for Biotechnology Information databases revealed 274 coding region variations have been documented in 1125 *TALDO1* sequences relative to 25 variations in 2870 *TKT* sequences (*P* < .0001). These findings suggest an unexpected prevalence and variety of genetic changes in human *TALDO1* with relevance for liver injury that may be preventable by treatment with NAC.

## 1 INTRODUCTION

Transaldolase (TAL) is a key enzyme in the non‐oxidative phase of the pentose phosphate pathway (PPP).^1^ TAL regulates the metabolic flux through the PPP and its output of NADPH as well as intracellular glutathione (GSH) levels, and thereby controls redox‐dependent survival and death signal processing in a cell type‐specific manner.^2^, ^3^, ^4^, ^5^, ^6^, ^7^, ^8^, ^9^ It is encoded by the *TALDO1* genomic locus that harbors an embedded repetitive element.^1^, ^10^ While a nearly complete but nonfunctional second pseudogene copy, TALDOP1, has been found on chromosome 1 in humans,^11^ the functional gene is encoded by a single genomic locus, designated as *TALDO1* on chromosome 11 at p15.5 in humans^11^ and on chromosome 7 in mice.^7^

Homozygous sequence variations of *TALDO1* locus have been mainly documented in children born to consanguineous parents^12^, ^13^ with a variety of clinical manifestations, including liver failure and cirrhosis, hepatosplenomegaly, growth retardation, developmental malformations, skin disease (eg, cutis laxa, dryness or ichthyosis, teleangiectasia, and hemangiomas), congenital heart disease, pancytopenia, bleeding tendency, immunodeficiency,^14^ hepatopulmonary^15^ and hepatorenal syndromes.^16^ In fibroblasts (FB) and lymphoblasts (LB) of the first TAL‐deficient patient, deletion of S171 (TALΔS171), an invariant residue located near catalytic K142,^17^, ^18^, ^19^ resulted in mis‐folding, proteasome‐mediated degradation and complete deficiency of enzymatic activity.^20^ Cells of this patient exhibit mitochondrial dysfunction, increased susceptibility to H_2_O_2_‐induced apoptosis and resistance to Fas/CD95 death receptor‐induced apoptosis.^6^

In comparison to wild‐type littermates (TAL^+/+^), mice partially (TAL^+/−^) or completely deficient of TAL (TAL^−/−^) exhibit liver cirrhosis and markedly increased rates of hepatocellular carcinoma (HCC).^8^ Homozygous (TAL^−/−^) and heterozygous TAL‐deficient mice (TAL^+/−^) are predisposed to hepatocyte necrosis induced by acetaminophen (APAP), which is the leading cause of acute liver failure in the United States^21^ and Europe.^22^ HCC and APAP‐induced liver failure were prevented by treatment with N‐acetylcysteine (NAC), an amino acid precursor of GSH.^8^ APAP toxicity is also attributed to GSH depletion and often responds to NAC treatment in human subjects.^23^ Nevertheless, the causes of APAP susceptibility remain unknown in most patients.^21^

The phenotype of TAL‐deficient mice clearly shows that this PPP enzyme plays a role in oxidative stress of hepatocytes and its deficiency accounts for liver disease of variable severity in children carrying inactivating variations.^13^ A unique substrate of TAL, sedoheptulose 7‐phosphate (S7P), is only accumulated in the urine of mice^7^, ^8^, ^24^ and humans with homozygous enzyme deficiency^25^ which has been the basis of screening for variations in TAL in newborns and children with unexplained liver failure.^13^, ^26^ However, the prevalence of heterozygous or homozygous TAL deficiency has not been examined in healthy adults or patients with predisposition to liver failure, such as survivors of APAP toxicity. Here, we report heterozygous TAL deficiency in peripheral blood lymphocytes (PBL) of two of 94 healthy adult Caucasian blood donors due to novel variations in the coding sequence, causing Q59Ter and S75N/94Ter and functional inactivation of the enzyme. We also noted variations that failed to affect enzymatic activity in a third healthy subject. Importantly, inactivating heterozygous variations were detected in DNA from four of 27 subjects with APAP‐induced liver failure. We also document two new cases of complete TAL deficiency due to (a) C→T variation at nucleotide position 610 of the TAL cDNA which resulted in amino acid S187F substitution in a child born to consanguineous parents of Turkish origin and diagnosed with liver cirrhosis at 7 months of age; and (b) insertion of a guanine (G) nucleotide at cDNA base position 103 which resulted in frame‐shifting and termination codon at amino acid position 81 in a child transplanted for congenital liver fibrosis (CLF) at 13 years of age in the United States. When comparing sequences deposited in the National Center for Biotechnology Information (NCBI), a surprisingly high frequency of coding sequence variations was found in *TALDO1* with respect to another enzyme in the non‐oxidative branch of the PPP, transketolase (*TKT*). These findings suggest that genetic screening for *TALDO1* variations, especially in populations at risk, may be relevant for preventing APAP‐induced liver injury and long‐term complications such as cirrhosis and HCC.

## 2 MATERIALS AND METHODS

### 2.1 Human subjects

PBL were isolated from 94 Caucasians medical and graduate student volunteers (46 males, mean age 23.4 years; 48 females, mean age 24.9 years) without any history of liver disease. PBL was used to prepare protein lysates in 40 mM tri‐ethanolamine (pH 7.6) and 5 mM EDTA (TEA/EDTA buffer) (a) to measure enzymatic activity and expression of TAL by western blot^1^, ^2^ and (b) to extract RNA for synthesis of cDNA and sequencing of the human TAL coding sequence. Genomic DNA was obtained from (a) 10 children with unexplained liver failure diagnosed in the Department of Pediatrics of Semmelweis University in Budapest, Hungary (four males, mean age 15.4 years; six females, mean age 11.0 years); and (b) a 7‐month‐old Turkish female patient with liver cirrhosis, who was diagnosed at the University of Ankara in Ankara, Turkey. Frozen tissue sections were received from 15 subjects, who have undergone liver transplantation, through the Liver Tissue Cell Distribution System located at the University of Minnesota (LTCDS, http://www.peds.umn.edu/gi/ltcds/).Genomic DNA samples were also analyzed from 27 subjects with liver failure upon exposure to >10 g/d APAP, which had been obtained through an Ancillary Study for the Acute Liver Failure Study Group hosted at the University of Texas Southwestern Medical Center in Dallas, Texas. Informed consent in writing was obtained from each subject, and the study protocol conformed to the ethical guidelines of the 1975 Declaration of Helsinki as reflected in a priori approval by the appropriate institutional review committee.

### 2.2 Detection of sequence variations in *TALDO1*

Total cellular RNA was extracted from peripheral from PBL or frozen tissues (Qiagen, Valencia, California), 3 μg of RNA was reverse transcribed to cDNA with oligo dT primers using 200 U of Superscript reverse transcriptase, according to the manufacturer's protocol (Invitrogen Life Technologies, Grand Island, New York). The TAL cDNA (GenBank Accession No: L19437.2) was amplified with 4/2BamHI/*BglII and* 4/1 Rev 2 primers.^17^ The coding region of the *TALDO1* chromosomal locus (GenBank accession number AF058913^10^) at 11p15.5^11^ was amplified with exon‐specific primer pairs, as described in the Supplemental Methods Section and shown in Figure S1). Polymerase chain reaction products were sequenced directly or cloned into the TA cloning vector pCR2.1 (InVitrogen) and sequenced in both strands. Nucleotide deviations from fully functional wild‐type TAL^1^ have been described as variations.^27^ The term mutation was reserved to sequence changes generated by site‐directed mutagenesis.

### 2.3 Site‐directed mutagenesis and expression of recombinant TAL

The TAL cDNA was cleaved with BglII and ligated into the BamHI site of the pGEX‐2 T expression vector.^17^ The plasmid was propagated in *Escherichia coli* strain JM105, while expression of recombinant protein was induced with 1 mM isopropyl‐β‐D‐thiogalactoside (IPTG) in the protease‐deficient BL21 strain of *E. coli*. The recombinant protein was affinity‐purified via binding of glutathione‐S‐transferase (GST) to GSH‐agarose beads that was followed by cleavage of TAL from bead‐bound GST using thrombin.^17^

### 2.4 Measurement of TAL expression

Enzymatic activity of TAL was measured in the forward and reverse reactions.^2^ Protein levels within PBL or liver tissue lysates were assessed relative to β‐actin by western blot.^5^, ^8^

### 2.5 Statistical analyses

Differences between continuous variables, such as enzymatic activities of wild‐type and mutant TAL proteins were analyzed by two‐tailed *t* test. Differences in categorical variables, such as frequencies of TAL variations in patient populations, were compared with two‐tailed chi‐square or Fisher's exact test using Prism Graphpad software (San Diego, California).

## 3 RESULTS

### 3.1 TAL variations in healthy control subjects

**Figure 1:**
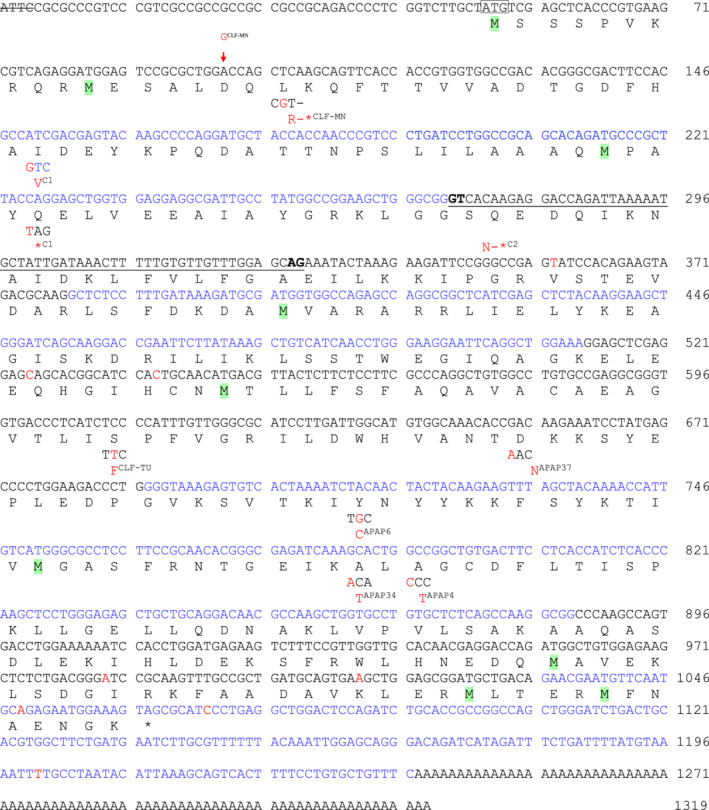
Variations in the TAL coding sequence and resultant amino acid changes that affect enzymatic activity of the translated protein. The nucleotide sequence is based on the TAL cDNA with a GenBank accession number L19437.2,^1^, ^10^ which is encoded by the *TALDO1* locus in region 15.5 of chromosome 11 with GenBank accession number AF058913.^11^ The wild‐type cDNA is comprised of 1319 nucleotides that encode 337 AMino acids, with the first methionine (M) being at base position 51. In the two healthy control subjects (C1 and C2) with reduced TAL activities, C → T transition at nucleotide position 225 and 59‐base deletion between positions 272‐330 (deleted nucleotides are underlined; potential alternative splice donor GT and splice acceptor AG sites are bolded) resulted in early termination codon at amino acid positions 39 and 94, respectively. In two subjects with congenital liver fibrosis from Minnesota (CLF‐MN) and Turkey (CLF‐TU), an insertion of nucleotide G after base position 102 resulted in a frame shift at amino acid position 18 (L18R) and a termination codon at position 81 and a C610T point variation causes serine to phenylalanine substitution at amino acid residue 187 (S187F), respectively. In four subjects with APAP‐induced liver failure, single nucleotide polymorphisms caused amino acid substitutions D202N, A246T, Y221C, and A248P

TAL expression was examined by western blot analysis of protein levels and measurement of enzymatic activity in PBL of 94 Caucasians medical and graduate student volunteers (46 males, mean age 23.4 years; 48 females, mean age 24.9 years) without any history of liver disease. TAL‐coding cDNA derived from the *TALDO1* genomic locus at chromosome 11p15.5^11^ was sequenced in all donors. Heterozygous variations were noted in four donors, C1‐C4 (Figure 1; Table 1). The mean ± SEM of TAL enzymatic activity was 36.4 ± 1.8 mU/mg protein across all donors. Controls C1 and C2 exhibited >50% diminished protein expression. C1 (18.2 ± 2.7 mU/mg protein, *P* = .020) and C2 also had diminished enzymatic activities (22.6 ± 4.8 mU/mg protein, *P* = .043). In donor C1, a C→T transition was noted at nucleotide position 225 which resulted in an early termination codon at amino acid positions 39 (Figure S2). Donor C1 also had an A→G transition at nucleotide position 194, which caused I29V amino acid substitution (Figure S2). In donor C2, a 59‐base deletion between positions 272 and 330 resulted in early termination codon at amino acid positions 94 (Figure S3; Table 1). The deletion of nucleotides 272‐330 involved exon 3 and it is flanked by typical GT splice donor and AG splice acceptor sites (Figures 1 and S3).^31^ A third control subject with normal TAL activity (38.9 ± 2.0 mU/mg protein), C3, had heterozygous variations at four nucleotide positions in the same strand, each causing amino changes (Figure S4A‐C). In the other strand of C3, a C→G transition was noted at nucleotide position 525, which resulted in a change at residue 159 of functionally similar Q→E amino acids (Figure S5). A fourth subject, C4, had reduced enzymatic activity (24.5 ± 1.9 mU/mg protein) and carried two variations in the 3′ untranslated region: C→T transition at nucleotide position 1070 and T→C transition at nucleotide position 1201 (Figures S6A,B). It is presently unclear whether these variations in the 3′ UTR affect gene expression by affecting RNA stability or translation. Thus, 20 new variations were uncovered in healthy subjects (Table 1).

**Table 1:** Sequence variations detected in the human *TALDO1* gene. Nucleotide and amino positions correspond to full‐length cDNA (GenBank Accession No: L19437.2)^1^, ^10^, ^17^

Variations (alleles)	Diagnosis/age/gender	AA change	Consequence
194 A → G; 225 C → T (1)	C1/22/F	I29V;Q39^a^	Inactivation^b^; (Figure S2)
272‐330 deletion (1)	C2/20/M	S75 N;94^a^	Inactivation; (Figure S3)
358 T → C,714A → T,984A → G,1012A → G (1)	C3/19/F	V63A,N222Y,I312V,K321R	(Figure S4A‐C)
525 C → G (1)	C3/19/F	Q159E	(Figure S5)
1070 C → T (1); 1201 T → C (1)	C4/22/F	None, 3′LTR	(Figure S6A,B)
103 insertion of G (2)	LF/13/M	L18R;Q81^a^	Inactivation; (Figure S10)
610 C → T (2)	LC/7 m/F	S187F	Inactivation; (Figure 11)
654 G → A (1)	APAP/37/M	D202N	Reduction^c^; (Figures 2 and S7)
786 G → A (1)	APAP/30/F	A246T	Reduction; (Figures 2 and S8)
712 A → G (1)	APAP/34/M	Y221C	Reduction; (Figures 2 and S9)
792 G → C (1)	APAP/22/M	A248P	Reduction; (Figures 2 and S9)
561‐563 deletion (2)	LC/9/F; LF/<1/M;	ΔS171	Inactivation^12^, ^28^
562 C → T (2)	HCC/16 m/M, LC/30 m/M	S171F	Inactivation^29^
624 C → T (2)	LF/2/M	R192C	Inactivation^28^, ^30^
625 G → A (2)	LF/<1/F	R192H	Inactivation^28^
895‐893 del (1)/931 G → A (1)	LF/<1/M	ΔN299/ G311R	Inactivation^14^

Abbreviations: Diagnoses: AA, amino acid, using single‐letter codes; APAP, APAP‐induced liver failure; C1‐C5, controls 1‐5; F, female; LC, liver cirrhosis; LF, liver failure; LS, liver steatosis; M, male.

a Termination codon.

b Inactivation refers to complete elimination of enzymatic activity.

c Reduction refers to a significant decrease of enzymatic activity relative to wild‐type transaldolase.^1^

### 3.2 Increased frequency of heterozygous inactivating variations in patients with APAP‐induced liver failure

**Figure 2:**
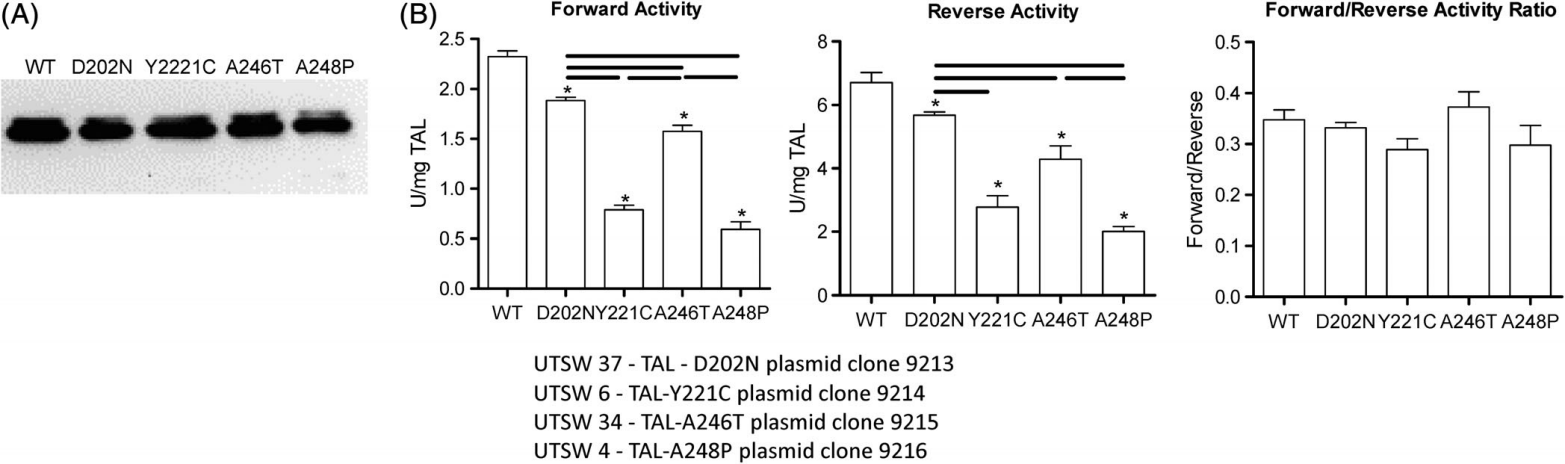
Reduced enzymatic activity of TAL in patients with APAP‐induced liver failure. A, Western blot detection of recombinant TAL with amino acid substitutions D202N, A246T, Y221C, and A248P. The functional consequences of these genetic variations were individually regenerated by site‐directed mutagenesis of recombinant human TAL cDNA cloned into the pGEX‐2 T expression vector. The presence of intended mutations and the absence of unintended mutations were confirmed by sequencing of both strands of the expression vectors. Recombinant proteins were expressed in *Escherichia coli*, affinity purified by binding to glutathione‐coated agarose beads, and cleaved by thrombin from GST in parallel with wild‐type TAL. B, Enzymatic activities of wild‐type and mutated recombinant TAL proteins were tested in parallel in three independent experiments. *P < .05 when comparing individual mutant proteins to wild‐type TAL; horizontal lines reflect differences in enzymatic activities between mutant proteins at *P* < .05

TAL deficiency predisposes to APAP‐induced liver failure in mice.^8^ Therefore, the entire coding region of the *TALDO1* locus was sequenced in 27 genomic DNA samples of human subjects with APAP‐induced liver failure. As shown in Figures S7‐S9, non‐synonymous, heterozygous variations were noted in four subjects, which resulted in the following amino acid substitutions: D202N, A246T, Y221C, and A248P. These patients (two 37‐year‐old Caucasian males, a 30‐year‐old Caucasian female, and a 22‐year‐old Asian male) had extremely elevated aspartate amino transferase (AST), alanine amino transferase (ALT) and bilirubin values, clearly indicating liver failure (Table S3). All patients promptly received intravenous NAC and fully recovered from liver failure. No primary cells or tissue were available to measure enzymatic activity in these subjects. Therefore, to test the functional consequences of these variations, each of them was individually regenerated in recombinant human TAL cDNA cloned into the pGEX‐2 T expression vector. The presence of intended variations and the absence of unintended variations were confirmed by sequencing of both strands of the expression vectors. Each of the variations noted in subjects with APAP‐induced liver failure reduced the enzymatic activity both in the forward and reverse reactions (Figure 2). Thus, the frequency of variations that limited enzymatic activity was higher in patients with APAP‐induced liver failure (4/27; 14.8%) in comparison to healthy subjects (2/94; 2.1%; Fisher's exact test *P* = .022).

### 3.3 Nucleotide insertion at base position 103 in the TAL coding sequence causes frame shift and early termination codon in a child with liver fibrosis in the United States

Complete TAL deficiency has been reported almost exclusively in children born to consanguineous parents of Middle‐Eastern, mostly of Turkish and Arabic origin.^13^ Two brothers with TAL deficiency, who were born to consanguineous parents, were also found in Poland.^28^ To assess the prevalence of TAL deficiency in CLF patients in the United States, frozen tissue sections were received from subjects that have undergone liver transplantation through the Liver Tissue Cell Distribution System located at the University of Minnesota (LTCDS, http://www.peds.umn.edu/gi/ltcds/). One of 15 tissue samples had undetectable TAL enzymatic activity relative to normal liver tissues with TAL activities of 13.7 ± 1.5 mU/mg protein. The cDNA prepared from this tissue carried a guanine (G) nucleotide insertion at position 103 of the coding sequence (Figure S10), which resulted in a frame shift at amino acid position 18 (L18Q) and a termination codon at position 81 (Figure 1 and Table 1). We also analyzed genomic DNA from 10 Hungarian children with liver failure, none of which contained variation in the promoter or the coding region, eight exons, of the *TALDO1* genomic locus (data not shown).

### 3.4 C610T nucleotide substitution causes serine to phenylalanine substitution (S187F) and inactivation of TAL in a Turkish female with liver cirrhosis

The patient is the first child of healthy, consanguineous Turkish parents. She was born after an uncomplicated pregnancy of 34‐week duration. She had a birth weight of 2400 g. At the age of 2 months, she presented with vomiting and intermittent diarrhea. Within several months, she developed hepatosplenomegaly. Her mental and motor development has been normal. She was hospitalized three times for lower respiratory tract infection at the age of 3 months. Family history was unremarkable. Physical examination revealed hepatosplenomegaly, 3/6 systolic heart murmur over the pulmonary heart valve. Her weight and height were 5900 g (3%‐10%) and 63 cm (25%‐50%), respectively.

Abnormal laboratory findings included hemoglobin of 9.8 g/dL, white blood cell count of 8300/mm^3^, platelet count of 201 000/mm^3^, AST of 96 U/L, ALT of 35 U/L, gamma‐glutamyl transferase of 43 U/L, alkaline phosphatase of 351 U/L, total bilirubin of 0.6 mg/dL, conjugated bilirubin of 0.3 mg/dL, prothrombin time of 16.2 seconds, partial thromboplastin time of 48.5 seconds, and international normalized ratio of 1.41. Other biochemical measures were within normal limits.

The serologic studies for viral hepatitis A, B, C, D, cytomegalovirus, Epstein‐Barr virus, rubella, and toxoplasmosis were negative.

Sweat test was normal. Thyroid function tests, α1‐antitrypsin level and α‐fetoprotein were normal. Metabolic investigations of her urine showed mildly elevated concentrations of carnitine and methionine. Concentrations of organic acids in urine revealed elevated concentrations of suberic acid (17.7. mmol/mol creatinine) and phenyllactic acid (149.15 mmol/mol creatinine; normal range, 0.03‐3.1). Arterial blood gasses, serum lactic acid, pyruvic acid, serum lipid levels, chitotriosidase, and urine glycosaminoglycans were normal. Reducing substance was negative in the urine. Electrocardiogram was normal. Echocardiogram revealed atrial septal defect. Spine X‐ray was normal. Ophthalmological examination was normal.

Abdominal ultrasonogram revealed hypertrophic left liver lobe and parenchymal heterogeneity. Portal system Doppler was normal.

A liver biopsy at the age of 7 months showed focal acinar transformation, hydropic degeneration, rare ballooning and multiple micro‐nodules with fibrosis, indicating micro‐nodular cirrhosis. There was no steatosis. There was no inflammatory infiltrate. Immunohistochemical staining provided no evidence of hemochromatosis and α1‐antitrypsin deficiency. Electron microscopy showed no specific findings for mitochondrial diseases.

She was referred for liver transplantation 2 months later. Two days after her admission, septic shock and pulmonary bleeding developed suddenly. Her blood culture became positive for Gram negative bacilli. Her platelet and neutrophil counts dropped, and she required multiple platelet and red cell transfusions. She was ventilated for 10 days because of poor respiratory effort. Her neutropenia and thrombocytopenia resolved gradually. In follow‐up, thrombocytopenia and mild neutropenia developed again. The neutropenia and thrombocytopenia gradually normalized within 15 days. Investigations for immunodeficiency were negative. By 9 months of age, her liver function tests have become normal. The early cirrhosis triggered investigation for TAL deficiency.

The patient's genomic DNA was examined for the TAL coding sequence in the *TALDO1* genomic locus at chromosome 11 p15.5 (GenBank: AF058913.1; http://www.ncbi.nlm.nih.gov/nuccore/7212866). As shown in Figure S11A, a homozygous C→T variation was found in exon 5 of *TALDO1*, which corresponds to nucleotide position 610 in the cDNA (GenBank Accession No: L19437.2). This variation results in a serine to threonine (S→F) substitution at amino acid position 187 (S187F). Urine or blood was unavailable for metabolite analysis to detect accumulation of S7P, which indicate a functional deficiency of TAL enzyme activity in mice^7^, ^8^, ^24^ and humans.^25^ Therefore, we mutated the wild‐type TAL cDNA in the pGEX2T expression vector (clone 1425) and created a recombinant TAL with a S187F substitution (TAL^S187F^, clone 9211). As shown in Figure S11B, the recombinant protein was expressed, purified, and tested for enzymatic activity. Unlike wild‐type TAL that was prepared in parallel (9.3 ± 1.1 U/mg protein), TAL^S187F^ had no enzymatic activity (0.05 ± 0.04 U/mg protein; *P* = .0007; Figure S11C).

### 3.5 Variations are more common in *TALDO1* than *TKT*

**Figure 3:**
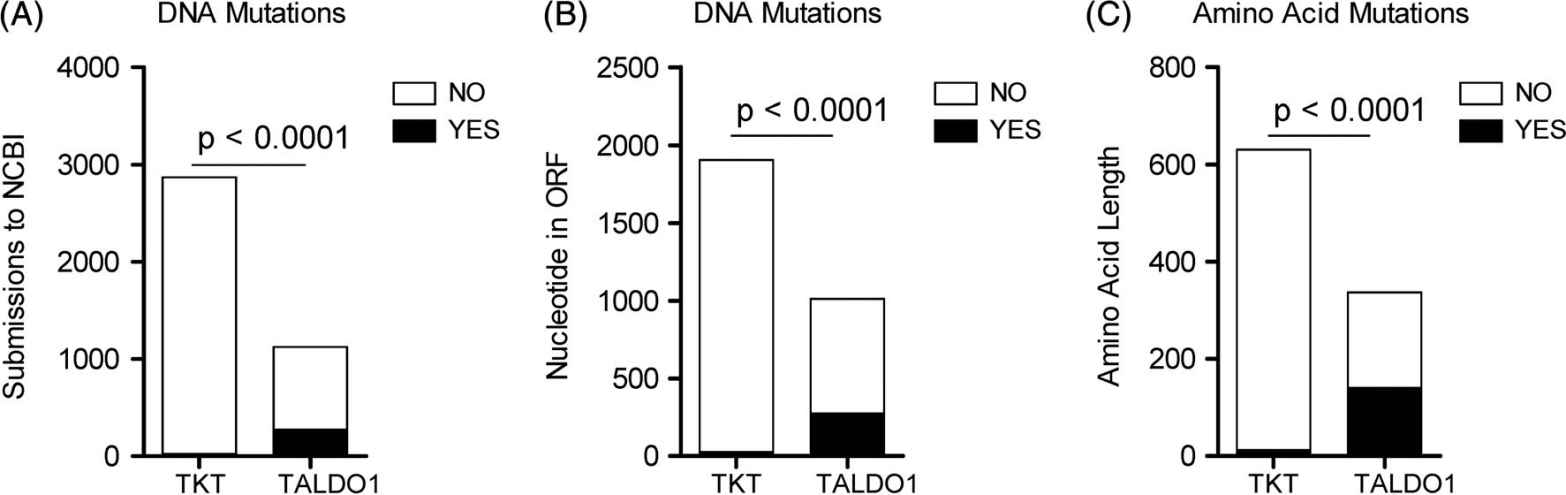
Frequency of variations in nucleotide sequences of TALDO1 and TKT submitted to NCBI. A, 274 variations were reported within the open reading frame (ORF) of *TALDO1* in 1125 human *TALDO1* sequences deposited in NCBI (http://www.ncbi.nlm.nih.gov/SNP/snp_ref.cgi?locusId=6888; Table S1). In comparison, 25 variations were reported within the ORF of *TKT* in 2870 sequences deposited in NCBI (http://www.ncbi.nlm.nih.gov/SNP/snp_ref.cgi?locusId=7086; Table S2). B, Bar chart representation of 274 coding sequence variations in the 1011‐nucleotide‐long ORF of *TALDO1* in comparison to 25 variations in the 1893‐nucleotide long ORF of *TKT*. C, Bar chart representation of 140 AMino acid changes documented within the 337 residue‐long peptide of TAL in comparison to detection of 12 AMino acid changes in the 631‐residue *TKT* protein. *P* values indicate differences using two‐tailed chi‐square analyses

Given the unexpectedly high frequency of *TALDO1* variations in our healthy cohort, we evaluated 1125 human *TALDO1* sequences deposited NCBI for sequence variations. As illustrated in Figures 3, 274 variations were reported within the 1011‐nucleotide long open reading frame (ORF) of *TALDO1* (http://www.ncbi.nlm.nih.gov/SNP/snp_ref.cgi?locusId=6888; Table S1). Of these 274 coding sequence variations in *TALDO1*, 140 resulted in amino acid changes within a 337 residue‐long peptide (Table S1). In contrast, only 25 variations were reported within an 1893‐nucleotide long coding region of 2870 human transketolase (*TKT*) sequences in NCBI (http://www.ncbi.nlm.nih.gov/SNP/snp_ref.cgi?locusId=7086) (Table S2). The frequency of variations in *TALDO1* markedly exceeded that in *TKT* with respect to the total numbers of sequence submission (Figure 3A) or the length of their coding sequences as reference points (Figure 3B). Furthermore, only 12 of the *TKT* variations resulted in amino acid changes within a 631‐residue protein (Table S2) relative to 140 amino acid changes documented in the 337 amino acid long coding region of *TALDO1* (Table S1). The frequency of amino acid changes reported in *TALDO1* also exceeded that in *TKT* (*χ* ^2^ = 693; two‐tailed *P* < .0001, Figure 3C). The mean number of variations, 24 per gene, upon sequencing 15 585 genes in 2440 individuals,^32^ was also exceeded by the frequency of coding sequence variations in *TALDO1*, 274 per 1125 submissions (*χ* ^2^ = 546; two‐tailed *P* < .0001).

As earlier documented, the *TALDO1* locus harbors a repetitive element, which is transcriptionally active due to the presence of an AluSc repetitive element with an RNA polymerase III promoter.^10^ Therefore, we enumerated the Alu elements within the *TALDO1* and *TKT* chromosomal loci. Using AluSc as a comparator, we allowed mismatches to detect all Alu elements having at least one complete left or right arm, as described earlier for *TALDO1*.^10^ In contrast to detecting 28 Alus in the 17479‐nucleotide‐long *TALDO1* locus, only 16 were found within the 40830‐nucleotide‐long *TKT* locus (two‐tailed *χ* ^2^ *P* = .0001). Alu sequences comprise 36% (6337/17479 nucleotides) of *TALDO1* locus, which is >3‐fold greater than their 11% overall contribution to the human genome.^33^

## 4 DISCUSSION

Complete, homozygous TAL deficiency has been previously associated with liver disease of varying severity that ranges from mild liver enzyme elevation^30^ to cirrhosis^28^ or complete organ failure at initial presentation^34^ (Table 1). TAL deficiency has been mainly documented in children born to consanguineous parents.^13^, ^29^, ^35^, ^36^ The scope of liver involvement in humans is reflected by the phenotype of mice with inactivated *TALDO1* locus.^8^ While the predisposition to HCC was initially documented TAL‐deficient mice,^8^ it was subsequently encountered in a human subject.^29^ Similar to humans with HCC, alpha‐fetoprotein (AFP) is also elevated in TAL‐deficient mice.^8^ Following its therapeutic efficacy in mice,^8^ NAC treatment was well tolerated and elicited clinical improvement, including the normalization of AFP in an infant with TAL deficiency.^37^

Importantly, complete deficiency and haplo‐insufficiency conferred similar predisposition to APAP‐induced liver necrosis in mice.^8^ As shown in the present study, the prevalence of TAL haplo‐insufficiency is increased among human subjects with history of APAP‐induced liver necrosis. Thus, TAL may indeed protect against to liver injury upon exposure to large doses of APAP. Notably, liver function has completely recovered in each of the four subjects with heterozygous TAL deficiency upon treatment with NAC. Recently, TAL deficiency was also reported in a 13‐month‐old male with mild liver failure and elevated AFP levels upon exposure to APAP, which were responsive to treatment with NAC.^38^

So far, 34 cases of complete TAL deficiency have been reported, which resulted in liver failure that required organ transplantation or led to death in most patients.^13^ Among the two children with complete TAL deficiency newly reported in this study, one with the S187F variation has recovered from liver failure. These findings may be connected to earlier observations that two of four siblings with the same inactivating variation also recovered from liver failure.^39^

Twenty new coding sequence variations of *TALDO1* are documented in this study (Table 1). A total of 274 variations have been annotated among 1125 *TALDO1* sequence submissions deposited in NCBI (Table S1). Three of the deposited *TALDO1* variations were also detected in our healthy cohort (525 C → G) and APAP cohorts (712 A → G and 654 G → A; Table 1 and Table S1). A higher copy number of Alu repeats, which are transcriptionally active,^10^ may be a source of increased sequence variation in *TALDO1*. Indeed, the mutability of a given gene or genomic region is influenced by non‐canonical (non‐B) secondary structures, such as Alu repeats, which can interfere with subsequent DNA replication and repair and increase variation frequency in a generalized fashion.^40^, ^41^ Moreover, the persistence of these variations in *TALDO1* may be connected to the overall dispensability of this enzyme for survival of individual cells or even the entire human organism.^9^ Unlike TAL, *TKT* activity is essential for survival of individual cells.^42^ Notwithstanding the relatively high, 5% prevalence of coding sequence variations in *TALDO1* in our healthy cohort, a complete deficiency of TAL is clearly uncommon. The relatively high frequency of sequence variations in *TALDO1* also indicate that most nucleotide changes may only have moderate effects on enzymatic activity without clinical consequences unless exposed to severe metabolic stress, as noted in four APAP‐induced liver failure patients. Large‐scale follow‐up studies are clearly warranted to further substantiate the significant prevalence of inactivating TAL variations in the general population as well as its role in predisposition to liver diseases, such as non‐alcoholic steatohepatitis, cirrhosis, HCC, and susceptibility to APAP‐induced liver failure.

The incidence of mating between two subjects that have a haploinsufficient allele frequency of 1/20 would be 1/400, and thus the incidence of homozygous null alleles would be 1/1600. However, complete TAL deficiency may affect male fertility^7^ and only half of the patients may live through childhood.^39^ Thus, according to a conservative estimate, less than 1/5000 young adults may have complete TAL deficiency. The prevalence of cirrhosis is ~400 000 or 15/10000 in the United States (http://digestive.niddk.nih.gov/statistics/statistics.htm). Since at least half of TAL‐deficient patients will have severe liver disease (cirrhosis or HCC), conservatively, TAL deficiency may account for liver disease in less than one of 30 (3.3%) patients. Via the accumulation of a signature substrate, S7P, complete TAL deficiency can be easily identified from as little as 100 μL of urine.^24^, ^25^ Although S7P does not accumulate in haplo‐insufficiency,^7^, ^8^, ^24^ a critical anti‐oxidant metabolite of the PPP, NADPH, is depleted in diseased tissues, such as the sperm^7^ and the liver.^8^ Due to the instability of NADPH and its lack of specificity, genetic screening offers the most reliable method of detection for partial TAL deficiency. Given the instability of S7P at ambient temperatures, genetic testing may also be the diagnostic method of choice in complete TAL deficiency when urine or mass spectroscopy laboratory is locally unavailable. As newly unveiled by this study, heterozygous TAL deficiency predisposes to APAP‐induced liver injury in human subjects similar to the mouse model.^8^ Importantly, NAC treatment prevents APAP‐induced liver injury as well as cirrhosis and HCC in TAL‐deficient mice.^8^ Based two promising cases of NAC efficacy in patients with TAL deficiency, genetic screening and early detection should be vigorously pursued to allow for such preventative intervention and to avert severe morbidities in human subjects. Therefore, prospective studies are warranted to determine the overall clinical efficacy of NAC in subjects with TAL deficiency.

## ETHICS STATEMENT

All procedures followed were in accordance with the ethical standards of the responsible committee on human experimentation (institutional and national) and with the Helsinki Declaration of 1975. Informed consent was obtained from all patients for being included in the study. This article does not contain any studies with animal subjects performed by the any of the authors.

## Supporting information


**Data S1**. Supplemental methods section.
**Table S1**. Variations in the open reading frame (ORF) of human transaldolase (TALDO1) documented in 1125 human sequences deposited NCBI.
**Table S2**. Variations of the open reading frame of human transketolase (TKT) documented in 2870 human sequences deposited NCBI.
**Table S3**. Demographic and clinical data of APAP‐induced liver failure patients with TAL mutations and partial loss of enzymatic activity.
**Figure S1**. Sequence of the TALDO1 genomic locus in region 15.5 of chromosome 11 (with GenBank accession number AF058913) (2).
**Figure S2**. Detection of mutations at nucleotide positions 194 (A→G) and 225 (C→T) in the TAL cDNA (GenBank Accession No: L19437.2) in a 22‐year‐old healthy female, C1.
**Figure S3**. Deletion of nucleotides 272‐330 deletion in TAL exon 3 of the cDNA in a 20‐year‐old healthy male, C2.
**Figure S4**. Detection of mutations at base position 358 (T6C, panel A), 714 (A6T, panel B), 986 (A6G, panel C), and 1012 (A6G, panel C) in the TAL cDNA (GenBank Accession No: L19437.2) of a 19‐year‐old healthy female, C3.
**Figure S5**. Detection of mutationat base position 525 (C6G) in the TAL cDNA (GenBank Accession No: L19437.2) of a 19‐year‐old healthy female, C4.
**Figure S6**. Detection of mutations at nucleotide positions 1070 (C6T, panel A) and 1201 (T6C, panel B) in the TAL cDNA (GenBank Accession No: L19437.2) of a 22‐year‐old healthy female, C5.
**Figure S7**. Mutation at base position 654 (G6A) in the TAL coding sequence (GenBank Accession No: L19437.2) of a 37‐year‐old male with APAP‐induced liver failure (UTSW37) in comparison to another subject with wild‐type sequence (UTSW38). Genomic DNA samples were sequenced directly.
**Figure S8**. Mutationat base position 786 (G6A) in the TALcoding sequence (GenBank Accession No: L19437.2) of a 30‐year‐old female with APAP‐induced liver failure (UTSW34) in comparison to another subject with wild‐type sequence (UTSW35). Genomic DNA samples were sequenced directly.
**Figure S9**. Mutationat base position 712 (A6G) in the TAL coding sequence (GenBank Accession No: L19437.2) of a 37‐year‐old male with APAP‐induced liver failure (UTSW6) as well as base position 792 (G6C) in a 22‐year‐old male with APAP‐induced liver failure (UTSW4).
**Figure S10**. Insertion of a guanine (G) nucleotide at base position 103 in the TAL cDNA (GenBank Accession No: L19437.2) of a 13‐year‐old male with congenital liver fibrosis (CLF). RNA was extracted and cDNA was prepared from frozen liver tissue and it was sequenced directly.
**Figure S11**.TAL deficiency is caused by an inactivating S187F mutation within the coding region of TALDO1 in a 7‐month‐old Turkish female patient with liver cirrhosis.
