# Supplementary material for: Transaldolase haploinsufficiency in subjects with acetaminophen‐induced liver failure
Source: J Inherit Metab Dis. 2020 Jan 1;43(3):496–506. doi: 10.1002/jimd.12197 (PMC7317976; doi:10.1002/jimd.12197)
Supplement: Supplementary file 1 — Data S1. Supplemental methods section. Table S1. Variations in the open reading frame (ORF) of human transaldolase (TALDO1) documented in 1125 human sequences deposited NCBI. Table S2. Variations of the open reading frame of human transketolase (TKT) documented in 2870 human sequences deposited NCBI. Table S3. Demographic and clinical data of APAP‐induced liver failure patients with TAL mutations and partial loss of enzymatic activity. Figure S1. Sequence of the TALDO1 genomic locus in region 15.5 of chromosome 11 (with GenBank accession number AF058913) (2). Figure S2. Detection of mutations at nucleotide positions 194 (A→G) and 225 (C→T) in the TAL cDNA (GenBank Accession No: L19437.2) in a 22‐year‐old healthy female, C1. Figure S3. Deletion of nucleotides 272‐330 deletion in TAL exon 3 of the cDNA in a 20‐year‐old healthy male, C2. Figure S4. Detection of mutations at base position 358 (T6C, panel A), 714 (A6T, panel B), 986 (A6G, panel C), and 1012 (A6G, panel C) in the TAL cDNA (GenBank Accession No: L19437.2) of a 19‐year‐old healthy female, C3. Figure S5. Detection of mutationat base position 525 (C6G) in the TAL cDNA (GenBank Accession No: L19437.2) of a 19‐year‐old healthy female, C4. Figure S6. Detection of mutations at nucleotide positions 1070 (C6T, panel A) and 1201 (T6C, panel B) in the TAL cDNA (GenBank Accession No: L19437.2) of a 22‐year‐old healthy female, C5. Figure S7. Mutation at base position 654 (G6A) in the TAL coding sequence (GenBank Accession No: L19437.2) of a 37‐year‐old male with APAP‐induced liver failure (UTSW37) in comparison to another subject with wild‐type sequence (UTSW38). Genomic DNA samples were sequenced directly. Figure S8. Mutationat base position 786 (G6A) in the TALcoding sequence (GenBank Accession No: L19437.2) of a 30‐year‐old female with APAP‐induced liver failure (UTSW34) in comparison to another subject with wild‐type sequence (UTSW35). Genomic DNA samples were sequenced directly. Figure S9. Mutationat base position [file JIMD-43-496-s001.pdf]

SUPPLEMENTAL MATERIALS

SUPPLEMENTAL METHODS

SUPPLEMENTAL TABLES S1-S3

SUPPLEMENTAL FIGURES S1-11

SUPPLEMENTAL METHODS SECTION

Description of synthetic oligonucleotide primers

Primers to detect TALDO1 cDNA:GenBank Accession No: L19437.2 (1)

4/2BamHI/*BglII* 57-74:
GTACTT*AGATCT*(-5′-)ATGTCGAGCTCACCCGTG-3'

Exon 8 Antisense primer (4/1 Rev 2)
5'- CAGCACAGGAAAAGTGACTGC -3'

Primers to detect the TALDO1 genomic locus: GenBank accession number AF058913 (2)

S1: Promoter and exon 1 sense (1FP_1)-369 to -346: 5'-ccagtcctggccccaccatggatc-3'
A1: Exon 1 antisense (1RP_1)232-252: 5'-caccgccacccggtccgcgtc-3'

S2: TALH exon2 sense primer 8357-8376: 5'-CGTCCTGGGGAATTACAGGG-3'
A2: TALH exon2 antisense primer 8604-8628: 5'-AAGGCCAACTAGACTAGCACGAGGG-3'

S3: TALH exon3 sense primer 11401-11428: 5'-CCTCATGATCCGCCCACCTCGGCCTCCC-3'
A3: TALH exon3 antisense primer 11761-11790: 5-GGAGCTCTTGGTCATCACTACCAACTGTGG-3

S2: TALH exon4 sense primer 12602-12626: 5′-GGCAGACACCCGGCCTCCAGCTTGC-3′
A4: TALH exon4 antisense primer 12912-12937: 5'-CTGTGCTCCCCAGTCTGGCATTCCCC-3'

S5: TALH exon5 sense primer 15773-15795: 5'-CCCCGCCCTCACCTGCCCCGCCC-3'
A6: TALH exon6 antisense primer 16459-16436: 5'-GGCACGGCAGTGCTGGCCTTGCCC-3'

S7: TALH exon7 sense primer 16684-16704: 5'-AAGGGGCCAAGGTGGGCAGGG-3'
A7: TALH exon7 antisense primer 16945-16925: 5'-CAGGCCCGGGCTCTCATAGGG-3'

S8: TALH exon8 sense primer 17241-17261: 5'-ACA CAG CTC GTG CTC TGT TTG -3'
A8: TALH exon8 antisense primer 17455-17475: 5'-CAG CAC AGG AAA AGT GAC TGC -3'

Reference List

1. Banki K, Halladay D, Perl A. Cloning and expression of the human gene for transaldolase: a novel highly repetitive element constitutes an integral part of the coding sequence. J Biol Chem 1994; 269:2847-2851.

2. Banki K, Eddy RL, Shows TB, Halladay DL, Bullrich F, Croce CM, et al. The human transaldolase gene (TALDO1) is located on chromosome 11 at p15.4-p15.5. Genomics 1997; 45:233-238.

Table S1. Variations in the open reading frame (ORF) of human transaldolase (TALDO1) documented in 1125 human sequences deposited NCBI (http://www.ncbi.nlm.nih.gov/SNP/snp_ref.cgi?locusId=6888). Out of 274 coding sequence variationsin the 1011-nucleotide long ORF, 140 resulted in amino acid changes within the 337 residue-long protein. Table S1 was also attached in Excel format. Coversion to pdf resulted in 28 consecutive pages on Hepatology website. **The original data can be accessed at the above NCBI website.**

Table S2. Variations of the open reading frame of human transketolase (TKT) documented in 2870 human sequences deposited NCBI (http://www.ncbi.nlm.nih.gov/SNP/snp_ref.cgi?locusId=7086). Out of 25 coding sequence variations in the 1893-nucleotide long ORF, 12 resulted in amino acid changes within the 631 residue-long protein. Table S2 was also attached in Excel format. Coversion to pdf resulted in 3 consecutive pages on Hepatology website. **The original data can be accessed at the above NCBI website.**

Table S3. Demographic and clinical data of APAP-induced liver failure patients with TAL mutations and partial loss of enzymatic activity.

Subject	Age (years)	Gender	Ethnicity	Bili	AST/ALT	Treatment	Outcome
UTSW 4	22	Male	Asian/non-Hispanic	8.8	2590/4305	NAC	Alive
UTSW 6	37	Male	White/non-Hispanic	9.7	1477/2289	NAC	Alive
UTSW 34	30	Female	White/non-Hispanic	2.7	6933/5417	NAC	Alive
UTSW 37	37	Male	White/non-Hispanic	4.8	8085/11660	NAC	Alive

LEGENDS TO SUPPLEMENTAL FIGURES

Fig. S1. Sequence of the TALDO1 genomic locus in region 15.5 of chromosome 11 (with GenBank accession number AF058913) (2).

Fig. S2. Detection of mutations at nucleotide positions 194 (A→G) and 225 (C→T) in the TAL cDNA (GenBank Accession No: L19437.2) in a 22-year-old healthy female, C1. The substituted residues are marked by arrows, blue in the wild-type sequence and red in the mutant sequence, respectively. The mutationwas confirmed by sequencing of the template after cloning of the heterozygous allele into the pCR2.1 vector (Invitrogen, Grand Island, NY).

Fig. S3. Deletion of nucleotides 272-330 deletion in TAL exon 3 of the cDNA in a 20-year-old healthy male, C2. The wild-type (top) and mutated (bottom) cDNA sequences are aligned. The deletion is flanked by typical GT splice donor and AG splice acceptor sites. The deleted nucleotides are underlined in the 5' end of exon 3 within the TAL cDNA in Figure 1.

Fig. S4. Detection of mutations at base position 358 (T→C, panel A), 714 (A→T, panel B), 986 (A→G, panel C), and 1012 (A→G, panel C) in the TAL cDNA (GenBank Accession No: L19437.2) of a 19-year-old healthy female, C3.

Fig. S5. Detection of mutationat base position 525 (C→G) in the TAL cDNA (GenBank Accession No: L19437.2) of a 19-year-old healthy female, C4.

Fig. S6. Detection of mutations at nucleotide positions 1070 (C→T, panel A) and 1201 (T→C, panel B) in the TAL cDNA (GenBank Accession No: L19437.2) of a 22-year-old healthy female, C5.

Fig. S7. Mutation at base position 654 (G→A) in the TAL coding sequence (GenBank Accession No: L19437.2) of a 37-year-old male with APAP-induced liver failure (UTSW37) in comparison to another subject with wild-type sequence (UTSW38). Genomic DNA samples were sequenced directly.

Fig. S8. Mutationat base position 786 (G→A) in the TALcoding sequence (GenBank Accession No: L19437.2) of a 30-year-old female with APAP-induced liver failure (UTSW34) in comparison to another subject with wild-type sequence (UTSW35). Genomic DNA samples were sequenced directly.

Fig. S9. Mutationat base position 712 (A→G) in the TAL coding sequence (GenBank Accession No: L19437.2) of a 37-year-old male with APAP-induced liver failure (UTSW6) as well as base position 792 (G→C) in a 22-year-old male with APAP-induced liver failure (UTSW4). The mutation in the DNA of one subject was compared to the corresponding wild-type sequence in the DNA of the other subject. Genomic DNA samples were sequenced directly.

Fig. S10. Insertion of a guanine (G) nucleotide at base position 103 in the TAL cDNA (GenBank Accession No: L19437.2) of a 13-year-old male with congenital liver fibrosis (CLF). RNA was extracted and cDNA was prepared from frozen liver tissue and it was sequenced directly.

Fig. S11.TAL deficiency is caused by an inactivating S187F mutation within the coding region of TALDO1 in a 7-month-old Turkish female patient with liver cirrhosis. A) Detection of a C→T mutation at nucleotide position 610 in the TAL cDNA (GenBank Accession No: L19437.2). This mutation results in a serine to phenylalanine (S→F) substitution at amino acid position 187 (S187F). B) Expression of wild-type (clone 1425) and mutated recombinant TAL with a S187F substitution (TAL^{S187F}, clone 9211) as a GST fusion protein in E. coli. Recombinant proteins were separated by SDS-PAGE and stained with Coomassie brilliant blue. Lanes contain whole cell lysate with GST-TAL fusion protein (lane GST-TAL) and affinity purified GST (lane GST) and TAL after cleavage with thrombin (lane TAL). C) Enzymatic activity of wild-type TAL and mutated TAL^{S187F}were tested in parallel in three independent experiments. Unlike wild-type TAL (9.3±1.1 U/mg protein), TAL^{S187F} had no enzymatic activity (0.05±0.04 U/mg protein; p=0.0007).

Figure S1. Sequence of the TALDO1 genomic locusin region 15.5 of chromosome 11 (with GenBank accession number AF058913) (2).

```
Minimal promoter
Transcription start site
Introns amplified in genomic DNA around exons
Exons
Stop codon

      -620 tgatcagaat atacaaggaa ctctgcaagt cagcgatggt aagaagcagg gcagctgggc
      -560 ctcctacgcc tgcctcgccg ttcggtcccc cggacacctc tgggcttagg actcagggga
      -500 acagattccc agaagcaggt tccccgccgc gtccggcgcc cgggctctgc agccgccccc
      -440 tgctctgcct cctgcacccg cagggcgtcc cccgccgctc cctcccctgc ttcctgcagc
PS1   -380 ggcccccgac cccagtcctg gccccaccat ggatcctgca tcgccgggtt cggccctggc
      -320 gggttcagcc ccgcagagtc ggcacccggg ccaggtccat ctcctccagc cctcccggtg
      -260 cgtcccgcgg ggcaggaagg aggcgaggcc cagccgcccc gtgtcgtgca ggtgttttcc
      -200 cgggcccgtc cgcggcggct gctgaggacc tggggagacc cagcctgtag gatccgcagc
      -140 tgcggtgcgc ggccggcagt ggcgctcggg cttcgtcccc gggggcgggg cttcgtccag
       -80 gcgcgcaggg accagcgggc ctcgccctcc cgcgccgctt tccgattggc agccgcccgc
       -20 ggggcattgt gggccgtccg

         1 cgcgcccgtc ccgtcgccgc cgccgccgcc gcagacccct cggtcttgct atgtcgagct
        61 cacccgtgaa gcgtcagagg atggagtccg cgctggacca gctcaagcag ttcaccaccg
       121 tggtggccga cacgggcgac ttccacggtg aggacggcgc ggagcccggg cgcggcgcaa
A1     181 gcgccctcca gaggccccgg cgccccgatt tccccgggtc tcccgttccg ggacgcggac
       241 cgggtggcgg tgccccgggc ggctcgttcc gggaggaatg agcgcaggtg ccggatgttg
       301 gggccaggga cagccgtgag gggagcgggg cccgggtcgc ccgtgagagg cgcagggcca
       361 gcggcgaccg tgaggggaac ggggcccggg tcggccgtga gggagcaggg ccgggggcag
       421 ccgtgaggag ctggggtcag ccgtgagggg ctcggtgctg gggtcagcgg tgcgggcggt
       481 actgacgggt ccttgcgagc cgtggggcac gaagccggag ccccagcctc gccctccgcc
       541 cagggtgggg tcggctgcgc cctcagaccc cgcacccggg acgggctgtg ggcagcccgc
       601 gggagcccct ctgtgcacct tcatgggtcg aggcagcttt cagagccttt gagttcagga
       661 gagattcagg gtagagcttc gagctgactt ccacgtggcc agtttgtggt cagcgagatg
       721 caattttgtt gggctgatgt ctgcagagcg gggacatcag ctagctctga ccagaagctg
       781 ccagtgctgc catcgccctt gagccctgcg gtgcccagtg ggacaactcc gtgggcggca
       841 ggtgcccggc ctgctggaca gccaccgcca gacgcaccgg gtgggtaaaa gccggtgccc
       901 atgaaagcac agggttggta cactgttaaa tactggattt tcctgggctt gagttgttta
       961 cattggagag aacttggaaa atacaaagaa ggattatttt attaatactt agctcttctg
      1021 tcctctgcag tgcagagtag acgagccttg tgtctatgtt ttgttgagaa caagtaatat
      1081 tttttaagaa aatcatgaaa cgcttcatgg atgaatgctg cgagcaccta atacggaagt
      1141 gacttcctct cttttctgct ttctcatggt gacttgagaa acaggcttta attcctcttt
      1201 ggtaactgag taagggcatt gtttatctag cagagcctag agagtgaatt ggcatgtttg
      1261 gctgcttaac cataaaatat ggggattcag tgactggctt ggggtgccac agtctgctcc
      1321 ggacccctct ttgggagatc caaagctaga gctgtgaatc cctcacacac acagcccccc
      1381 cgttgtagtc cagacctctc ttcataagaa agaaagctcc agaaaagggg ctacacattt
      1441 ttctgctgct cagtttttc ccttcttagc actgagaata atttgcagtt tgcctggtgg
      1501 acagagcgct catctgtatt gttccttgct ggccctcacc agttagggca gaactggtgc
      1561 ggagtgtcag gtccattaca cttggcaatt caagggcaat aaagatggat tttcctaaac
      1621 ccacacccac aggagggctg gggagtcttg ggtttctggt tttgttttgt attgtttttt
      1681 aagaaaactt ttggcccagc gaggtggctc acgcctgtaa tcccagcact ttgggaggtt
      1741 gaggcaggtg gatcacgagg tcaggagttc gagaccagcc tgaccaacat ggtgaaaccc
      1801 cgtctctact aaaaatacaa aaattagctg ggtgcggtgt tgcatgcttg taatcccagc
      1861 tacttgggag gctgaggcag gagaattgct gaacccagaa ggcggaggtt gcagtgagcc
      1921 aagatcacac cactgcactc cagcctgggc gacagagcaa aactccgtct caaaaaaaaa
      1981 aaaaaaaaaa aatcttctaa acccaagttg tgacattttt ttttaatgaa tattaacaaa
      2041 aaaatacatt cattgatagt atatgttcat aaatgttata tagttgtaaa atagtctgtt
```

2101 gggtttgtga tctgtagttt gtgaagtgaa ggttcttaac tccttgtggg agggaggcac
2161 aggcctctgg aaatagaatc aaagctgtga atgctcttcc cacaatgtga tcctccccat
2221 tttgcattca gtttaaggat ccctggaccc tgaaacccat tttgccccca atgggccttg
2281 gttttgtttt aattttgtaa aagccatttg ctaaaagctg actttatcca ggggtgattt
2341 tacaggacga aatgggctgt tttcagtgcc tcattcagtg cattctggca gacctgtgtt
2401 agcactgtgc cagatgtcat gttgaaatga gtatagatgc acaacaggct ttttcttaac
2461 ttgtcatgtt gaaatgggta tagatgcaca gaagttgcaa aatcagccca tacagtcctg
2521 tgaacccttc aaccactgtc ccccatggtg acatctcatg tgaccatggc atggcatcca
2581 caccgggaag tgggcactgc tgtggtgctg atgctgttat agactgcagg cctggctcag
2641 ggctccccag tttcccccctg cactggggca aggaggctat gtgacagttt cataaagtgt
2701 gtaaattcct ggaccactgt gacaggacat agctgtccca ttgtcactga gatcccgtgt
2761 ccctcagaga cccctcattc cctgttcctt ggcaacctct aacctgtttt ctacctctat
2821 agttttgtca ttaaaggacc ctttgaaagt acttattcca gcctgcgcaa catggtaaaa
2881 ccccatctct acaaaaagga agaaaaaaaa ataccaagcg tattggtgca caccgtagtc
2941 ctagctattc aggaggttga gttgggagaa tcacttaagc cccaggaggt tgaggcagca
3001 gtgagcttgt gactgcgcca atgcactcca gcttaggaga caaagtgaga ccttgtctca
3061 aaaaaaaaaa aagatattta tttgccaaat aaagttactg aacttgtgaa cttcggccgg
3121 gtgtggtggc tcaagcctgt aatccgagca ctttgggagg ccgaggcagg ccgatcatga
3181 ggtcaggaga tccagaccat cctggctaac acggtgaaac cccatctcta ctaaaaatac
3241 aaaaaaatta tctgggcgtg gtggcgggcg cctgtagtcc cagctactcg ggaggctgag
3301 gcaggagaat ggtgcgaacc tgggaggcgg agcttgcaat gagccgagat cgcgccactg
3361 cactccagcc tgggcgacag agcgagactc tgtctcaaaa gaaaaaaaaa aagttactga
3421 actggctaat ctggagcaat gggccattct atgggtgtca atggccataa ctcttcacct
3481 ccactttcca tttggacagc cagatgaaca agaccccagg ttaagcctcg ttggcttctt
3541 tgaattccta gcttggttat gtgagaaata actaatgtgt taaaagaaac aatagtttta
3601 agaataaaga atcccttggg tttttgtttt tggggttttt ttggagacag ggtcttgctc
3661 tgtcacccag gctgtatagt ggtgcaatca cgtggtgcaa cacggctaac tgcagccttg
3721 acctcctggg ctgaggtgat ccccccacct cagcctccca agtagctggg actacagatg
3781 tgcgccacca ctcccagcta acaatccttg ctgaagataa ccatgtggta tctgtgttga
3841 agtcaaaata aaatgtagag acgaatctct aaattcaacg ttttatttgg gaagaaaaaa
3901 ttgcagttca agcagactgg gtggtcttcg atctgtccaa agaacaagag aaagttgggg
3961 ccagcctcgg tggctcatgc gtataatccc cagcactttg ggaggctgag gcagaaggat
4021 tgtttgggcc tagcagttcg ggaccagcct gggcaacaca gtgagaccac atctctacaa
4081 aaaatttttg gctggtggct tacgcctgta atcacagcac tttgggaggc cgaggagggt
4141 ggatcacctg aggtcaagag atcgagacca gcctggccaa catggtgaaa ccccatctct
4201 actaaaaata caaaaaatta gccgggtatg gtggcaggtg cctgtaatcc cagctactcg
4261 agaggctgag gcgagataat tgcttgaacc tggaagatgg aggttgcagt gagccgggat
4321 cacgccactg cactctagcc tgggcgacag agcgagactc catctcaaaa taacaataat
4381 aataataata aaccatcaac ctcattttga tggtaattga tgtgtgtgca tgcatgcatg
4441 tgtgtgtaca cagttcctgt ctcataactc ccacagccct gttataatgt tggggcactc
4501 taggcctcag gagcaggcct tggaaaacag aatttccatc actgaccttc tcctctcctt
4561 tcacctgttc ctttctctct tcaagggagg aatctttcct gcctgttttg gagctgtcca
4621 taaataaatt ctctacttgc ttttcttttc tttttttttg gaggcggagt ctcgctctgt
4681 cgcccaggct ggagtcagtg gcgtgatctc agctcactgc aagctccacc ttccgggttc
4741 acgccattct tctgcctcag cctccagagt agctgggata caggcgcccg ccaccacgcc
4801 cacctaattt tttgtatttt tttttttttt ttagtagaga tggggtttca ccgtgttagc
4861 caggatggtc tggatctcct gacctcgtga tctgcccgcc tcggcctcct aaagtgctgg
4921 gattacaggc accagccacc gcgcccggcc gtctacttgc ttttctaatt gtgggtccta
4981 agaccctcat ttcaaaaggg gtcccatcac ataccctgga ggaaggaatg ttgcacggag
5041 gacggtaaga aggtgaacac agagcgttgc tgggtttcct cactcattag atgataatat
5101 ttcaacaggg ctgtccatac ttcagtcatg tctctccagt agagtctcca taaaaggccc
5161 aagaggacag ggttcaggga gcttccaaat acctgaacac atggagcctc ctgaagggtg
5221 gtgcacccag ggtgggcatg gaggcaccgt gccccttctc acatgccttg ccctatgcat
5281 ctcttcctct gtgtctttgt ataataagcc agtacatgta agtgagtgtt tcccttactt
5341 ctgtgatctg ctccagcaaa ttaatggaac acaaaggtta attcaagttg acaaccccaa
5401 gttgaagcca gtggatcaga agttctggag cccagacttg tgacgggtgg gaatggagaa
5461 tggttggttg ggactaagcc ttcaacctgt gggatctgag gctgtctcca ggtagacagg

5521 gttggaattg aattggagga tgcccagctg gtatccactg aagaattaat tgcttgtttg
5581 gtgcgtgggg agaaaccccc acacatcggt cacagaagtc ctctctgttg attattctgg
5641 tatgagagca gaggaatgca gttcaggttg tttcttcaga ggccgggcgc agtggctcac
5701 gtctgtaatc ccagcaattt gggaggccaa ggtgggtgga tcacctgagg tcaggagttc
5761 gagaccagcc cggccaacat ggcaaaaccc cgtctctact aaaaatacaa aaaaaaaagg
5821 ccgggtgtgg tggctcacac ctgtaacccc agcactttgg gaggctgagg cgggcggatc
5881 atgaggtcag gagatcgaga ccatcctggc taacacggtg aaaccccatc tctactaaaa
5941 atacaaaaaa ctagccggcc gcagtggcgg gcgcctgtag tcccagctac tcgggaggct
6001 gaggcaggag aatggcgtga acccgggagg cagagcttgc agtgagccga gatggcgcca
6061 ctgcagtccg gcgtgggtga aagagcgaga ctccgtctca aaaaaataaa aatacaaata
6121 caaaaaaaaa aacacaaaaa accatagcct ggcgtggtgg ctcgtgcctg taatcccagc
6181 tactcatggg gctgaggcag gagaattact tgaacctggg agttggaggt tgcagtgagc
6241 tgagcttgcg ccactgcact ccagcctggg caacagtgca agactccgtc tcggaaaaaa
6301 ataaaaaata aatacaagta caaaaattag ccaggcatca tggtgtgagc ctgtagtccc
6361 agctactgag gacgctgagg taggagaatc acttgaaccc tggaggagga ggttgcagtg
6421 aggcgagatc attttactgc actccagcct ggatgacaga gtgagactct gtgtcagaaa
6481 aaaaaaaaaa gctactcagt ggctacaagt ttggacgttt tgaactgttt agttggttac
6541 tctgaagaaa aaaacttttt tattttttt tgcaacagag tcttgctcca ttgcccaggc
6601 tgtagtgcag tggcacaatc tcggctcact gcaacctcca cctcccgggt ccaagtgatt
6661 ttcctgccac agcctcccga gtagctggga ttataggcat gcaccaccac gcctggctaa
6721 tttttgtatt tttagtaggg acagggtttc gccgtgttag ccaggctggt ctcgaactcc
6781 tgacctcaag tgatccaccc ccgttggcct cccaaagtgc tgggattaca ggtgtgagcc
6841 actgtgccca gccactgagt agcattttat gactgggttg attaaagcag agaatgtcag
6901 ctttataaaa gggaccacca gtacaatttg aacaaaaact tgggacattg tcaaagttgt
6961 ccactccgac taaaaggatc ttaggtcagg ttttgtcaag ttacctgcag aagctactga
7021 atgcgatatt tatttatttg tttgcttatt tatttatgaa acagagtctc actctgtcac
7081 ccaggctgga gtgcagtggt gcaatctcag ctcattgcaa cctccacctc ctgggttcaa
7141 gcgattctct tgcctcagcc tcctgagtag ctgggattac gggcatgtgt caccacaccc
7201 agctcatttt tgtattttta gtagagactg ggtttcacca ttttggccag gctggtttca
7261 aactcctgac ctcaggcgat ccaccagcct cagcctccca aaatgctggg attacaggca
7321 tgagccactg cacccggcct gaatttattt ttttaagaga tagggtcttg ctctctggcc
7381 caggtgggag tgtagtggca tgatcatggc tcactgcagt ctcaacctcc tgggctcaag
7441 cgatcctcct gcctcagcct cttgagtagc tgacgctaca ggcatgcact accacacctg
7501 gctaatttag tctttctatg aaacctaaaa agaatgattg gtgagtgata gcagtggata
7561 aaattagtct taaagttgca gcagtgcggt tcagcaacta ttagataaac tggtttcagg
7621 ttagaggccg cttcagcaac taggcttgca ggtaataatt tttggagcaa tgctgtgtgt
7681 cctgagtgct tttccccttg gccttttttt tttttttttt tttttttttg agatggagtc
7741 tcactctgtc acccatgctg gagtgcagtg gcgcgatctc ggctcactgc aagctccgcc
7801 tactggattc aagccattct cctgcctcag cctcccgagt tagctgggac tacaggtgcc
7861 gaccaccaca cccagctagt tttttgtatt tttaataaag acggggtttc accgtgttaa
7921 ccaggatggt cttgatctcc tgacctcgtg atccacccgc ttcagcctcc caaagtgctg
7981 ggattacagg cgtgggccgc cgcgcccact ccccttggct tcttgactct gttgcagctg
8041 ggtatggtga taatgaccca gtccataaga tcagcgttca catgtggctc atgactagac
8101 cgcgcgcctc cttgtggtgg tacggcagtg ggatgcacat tttcctttga ccagcgttct
8161 cccagctgtc gcaccgctca ctcccatgct gttacaggtc acctcttgca ggggcatatt
8221 tgatcactgg gcacttagtg tttgttgttc catcattatg caaacagccg cctcttgatg
8281 aaccttcagt gacccagaag aaggtgtgag aagtgtggct ggaccccgct ttcggaaatg
S2 8341 ctctggactc ccctaacgtc ctggggaatt acagggttcc ttcacatgtt tccagttgga
8401 agcaagtact ccacttactt tggcttttga aaactatttc cctagccatc gacgagtaca
8461 agccccagga tgctaccacc aacccgtccc tgatcctggc cgcagcacag atgcccgctt
8521 accaggagct ggtggaggag gcgattgcct atggccggaa gctgggcggg tgagtgcctg
A2 8581 gactcgggta gggtccagct aggccctcgt gctagtctag ttggccttgc ttccctccct
8641 aactgaattt tagttctcaa acaccatgaa ctcaaggggg gaaaaaaacc ctactctttt
8701 tgcctatttt tgtttattga agtgtaacct gcatgaagta acctgcacct gtggtaaatg
8761 agcagttcag agagttttgc caatgtgtgt accctgtaaa taccacccca gtcaagatgc
8821 agagcacttg cagaccccca caggcccctc ctcccctcct gtagtcagtc tccccagctc
8881 tggtaacact taccttctgg ctgtcatttt attttttact tttcagacgg agtctcgctg

```
       8941 tgtcacccag gctggagtat agtggagcaa tcttggctca ctacaacttc cgcctccctg
       9001 gtgcaagcaa ttctcctgcc tcagcttccc aattagctgg gcttacaggt gtgtgccacc
       9061 actcctggct catttttgt atttttttt ttttttttt gagacagagt tttgcttttg
       9121 ttgtccaggc tggagtgcac tggcacaatc tcggctcacc gcaacctctg cctcccagat
       9181 tcaagcgatt ctcctgcctc agcctgccga gcagctggga ttacaggcat gcgccatcac
       9241 acctggctat aatttttgta tttttggtgg agacagggtt tcaccatgtt ggccaggccg
       9301 gtctcgaact tcttacctca agtgatccac ctgccttggc ctcccacagt gctcggatta
       9361 cagacgtgag ccaccgtgcc tggcttttta attttttaat agcttttata actcccagaa
       9421 tgttctctgg tacctgtttg cagtagctcc ccactccctt acccaaccaa gcaactgtag
       9481 agccctcttc atgcctgtgg ctttgcatat tttagatgtc attttagatt ctcttcaggg
       9541 taataattca aagcttgaag atggtgccga gcaggcaacg tgggcaccac acaggagcag
       9601 gtccctctct ggtactggct ttgcttaggt ccaggtggct ggtgagtggc tcccctgacc
       9661 atgttgtgac agccactctg gacactgagt ctgaccctct cctctctctt cttagctccc
       9721 ttttcctcat ctcgtggtgt tgcacccctg tggcatcagt gaggccttgg tgctcctgtt
       9781 attgggtgac attggtgcct ttgcttggct gggtgatcaa cagatgagcc ctgcacccat
       9841 gtccagatgg ccccaatttt ttttttttt tttttgagat agagtttggc tctgtcacct
       9901 aggctggagt gcagtgtcac aatctcagct cactgcagcc tctacatcct aggctcaagc
       9961 cagcctccca cctcagcctc ccaagtagct gggactacag gtgcacgcca ccacacctgg
      10021 ctaatatttg tatttgtttt tagaggcagg gtttcaccat gttgccctag ctggtttcga
      10081 actcctgagc ttgagcagtc tgcccacctt ggcctgccag ggtgctgtga ttacaggcgt
      10141 gagccactgc acccagtctg gcccaagaat tctaacctat gagcgtctag ctgaagatct
      10201 tcctgtgtgt agtgggctaa caaggctgct cagattttgg aacagaatca tggacttcac
      10261 tgggtgtctg tccaataact ttgaagagat agaaaatata atattttgca agtgccaaaa
      10321 gatagaggga tgcagtttgg gatgatgaaa agagctctgg agatggatgg tgatggttgt
      10381 acagaactgt gaatgtgctg aatacatttt aaaatggtgg gctgggggca gtggtgcacg
      10441 cctgtaaccc cagtgctttg gtaggccaag atgggaggat cccttcagcc taagagttcg
      10501 agaacagtct aggcaatagg gcaaaactct gtctctacaa aaaaatataa aaattagctg
      10561 ggcatgggcc aggcgtggtg gctcacgcct gtaatcccag cactttgaga ggccaagaca
      10621 ggcggatcac gaggtcagga gatcgagacc atcctggcta acacggtgaa accccgtctc
      10681 caccaaaaat acaaaaaaat tagccgggca cagtggcggg cgcctgtagt cccagctact
      10741 caggaggttg aggcaggaga atggcgtgaa ccgcgggggg cagagctggc agagccgaga
      10801 ttgtgccact gcactccagc ctgggcgaca gagtgacact ccatctcaac aaaacaaaaa
      10861 aattagccag gtatagaggt gtgcagctgt agtcccagct acttgggagg ctgaggcagg
      10921 aggatcactt gagcccggga ggtcaaggct gtaatgagcc ataatcacac tactgtgctt
      10981 ccagccttag ctggagaccc tgtcttgggg aaaaaaaaaa ggataagatg ggaaatttta
      11041 tgttatatgt attatttggc cacaataaaa aaatgtttta agagaaaaaa aaagatggag
      11101 ggaaggaaat ttggcaaaga aataacagaa tttattttgt agagtcttgt gcacttgaac
      11161 ctcagaagct tcttttttt tttcttgaga tggagtctcg ctctgtcgct caggctggag
      11221 tgcagtggca cgatctctgc tcactgcaag ctccacctcc caggttcacg tcattctcct
      11281 ccctcagcct ccctagtaac taggactaca ggcacccgcc accatgccca gctaattttt
      11341 taatattttt agtggagacg gggtttcacc gtgttagcca ggatggtctt gatctcctga
S3    11401 cctcatgatc cgcccacctc ggcctcccaa agtgctggga ttacaggcgt gagccaccgc
      11461 acctggcgaa cctcagaagc ttctaacctg ctttttccc tttgaatttc aggtcacaag
      11521 aggaccagat taaaaatgct attgataaac tttttgtgtt gtttggagca gaaatactaa
      11581 agaagattcc gggccgagta tccacagaag tagacgcaag gtaaggatgc ttgctcctgc
A3    11641 actggatggg ctggtcaggt gtccacagtt gcacacgtgg atgccaacat gaggtcaagg
      11701 ggctgctcca cccatggtct tcatcccaag ggcccaagag gaggtttatt ttgagctctc
      11761 ccacagttgg tagtgatgac caagagctcc ttgtttagca actgatctct agtgtttttt
      11821 atttttattt ttatttattt ttgagatgga gtctcgcact gtcacccagg ctggagtaca
      11881 gtggcgcaat ctcagctcac tgcaacctct gtctcccggc ttgaagcgtt tctctgcctc
      11941 agcctcccaa gtagctgaga ttacaggcac ctgccaccat gcccagctaa tttgttgtat
      12001 ttttaataga gatggggttt gaccattttg gccaggctgg tcttgaactc ctgacgtcat
      12061 gatccacctg cctcggcctc ccaaagtgct gggatcacag gcatgagcca ctgtgcccgg
      12121 ctgctagtgt tttttgtttt gatttgtttt gtttgttttt tgagacagag tctcgctctg
      12181 ttgccagtgt agagtgcagt ggtgtgatct cagctcactg caaccttcac ctcccgggtt
      12241 caagcgattc tcctgcctca gcctcccaag tagctgggac tacagacacc cgccaccata
      12301 cccagctaat ttttttgtat ttttagtagc gacagggttt caccatgttg gccaggatgg
```

12361 tctcgatccc ttgaccttgt gatcttcccg cctcagcctc ccaaagtgtt gggattacag
12421 gcgtgagcca cggcacctgg cccagtgttt tttaatacaa attatagcct atggatccca
S4 12481 ctgaggactc tgtttgtgag actgggtgtg agttgctgtc agagccattc tgcaaacctc
12541 cagtgagggg aaagttgagg gcagtggttg gtgctcacgg tggtgctgct caaaactgac
12601 aggcagacac ccggcctcca gcttgctctg cgtggtgggc aacctgcgtg atctgagggg
12661 atgggttctt cttgctccta caggctctcc tttgataaag atgcgatggt ggccagagcc
12721 aggcggctca tcgagctcta caaggaagct gggatcagca aggaccgaat tcttataaag
12781 ctgtcatcaa cctgggaagg aattcaggct ggaaagtaag tggtccccca caaggaagga
12841 gctctcagag atttttcctc tgttggagca gggtctacat ggcatcaggc aagtttgact
A4 12901 ctctcagccc tggggaatgc cagactgggg agcacagccc acagcttgga cttgaccagc
12961 tcatgtcagc ctagatctgc cctctgctcc agagccagct ccctcacttg ctggtgatca
13021 gaaatgaggg atttcccagg aagtaccatc cacccactca gcagagaaga aaagccacta
13081 gttagcacct gcagattcag attctgtcct tcctctgtcc tgatcaacac aagcctatgt
13141 ttacatgatt tttaaaaagt aaactattgt tatataaaat atatgctggc tgggcaagat
13201 ggctcatgcc tgtaatccca gcaatttggg aggctgagat gggaggatca cttgagccca
13261 ggagttccag accagcctgg gcaacaaagt gaaaccttgt ctctacaaaa atataaagaa
13321 attttaggct gggcacggtg gctcccagca ctttgggagg ccgaggtgtg gggatcacga
13381 agtcaggaga tcgagaccat cctggctaac acggtgaaac cccgactcca ctaaaaaata
13441 taaaaaatta gccgggcatg gtggcacaca cctgtagtcc catttaggag gctgaggcag
13501 gagaattgct tgaacccagg aagcgaaggt tgcagttagc tgagatcaca ccactgcatt
13561 ccagtctggg tgacagaaca agactctgtc tcaaaaagaa aagaaaactt ttaggccggg
13621 cacggtggct catacctgta atcctagcac tctggaagcc tgaggtgggt agatcacctg
13681 aggtcaggag ttcgagacca ggctggccaa gatagtggaa ccccgtctct accaaaacta
13741 caaaaaattt agctgggcgt ggtggcaggt gcctgtaatc ctagctacat gggaggctga
13801 agcaggagaa tcacttcaac ctggggaggt ggacgttgca gtgagccgag attgcaccat
13861 tgtactccag cctgggtgac agaatgagac tccatctcaa aaaaaaaaaa aaaaaagaaa
13921 agaaaaacta gcccacccct gtgattccag ctacttagga ggctgaggcg ggaggatcgc
13981 ttgagcctgg gaagttgagg ttccagtagc tatgatcgta ccactgcact ccagcctggg
14041 tgacagagtg agaccctgtc tcagaaaaac caaaccaaac atatgtatgt tcagagtgga
14101 agagacagaa aatgtataaa acaaagtaaa actcctctat gatgttctca cccagaagca
14161 gccaatgcta tactcaaata attcgcatca ggaagaggtg ttttgtttcc acatctgtgt
14221 gtctgcatgt tctaccacct gccatcactg acctgctctg agatagcatc tcactttttc
14281 accacggctg gagtgcagtg gcacgaactt ggctcactgc agccttgact ccaggctcaa
14341 gctatctttc cacctcagtc ccccaagtgg ctgggatgac aagcacacat accactcctg
14401 ggtaatttca ccacgttccc caggctggtc tcgaacccct gggctcaaga gatctctacc
14461 catcttggcc tcccaaagtg ctgggattat aggcatgagc cattgtgcct ggcctcattt
14521 gtgtgttttt ttgttttttt ttgtttttga gacggagttt cattctcgtc catgctggag
14581 tgcaatggtg ctatctcagc tcactgcaac ctccacctct ggggttcaag agattctcct
14641 gcctcagcct ccggagtagc tgggattaca ggcaggcgcc accactcccg cctaatttta
14701 tatttttagt aaagatgggg tttctcatgt tagtcaggct gatctcaaac tcccaacctc
14761 aggtgatccg cccgcctcgg cctcccaaag tgttgggatc acaggtttga gccactgcac
14821 ctggccggac catttgtttt tatatgttgg aaaatactcc attttatgga taggccacag
14881 ctattcatct gttggtggac acctggtcag tttccatctc ttgagctgtg atgtatattg
14941 ctgtgggcgg cttccatttc tcgggctgtg atgtatgctg ctgctctgaa cacatctgct
15001 gctatgaaca catctgctgc tctgaacaca gatgtgcaag ttttgtcctg gacatgtttt
15061 tctgggttct cgctgggcat atgcctagga gtggagtctc tgggtggact gctggaggaa
15121 ctgcagggtg tttcccaact caggtgtgcc atcttcgtgt ccccccctcga acaccccgtg
15181 ttggccattc gcagaggccc ctggggaggg aacttgtgtc tgaaggtgcc tgagcagagc
15241 cttggttgtc tcatcccatg ccccagccac atggcaaagt atgggcactg ctccgccctt
15301 ggggatcatc cccaggatgt ccaaagtgca ctgggatttg ggcaagtggc ctagtgactc
15361 caaagggggct cctctcagca gaaaccaggg tgctacgggc ctgctgcagg gtggccccag
15421 gaatgggtag ggtggaacag ggtgaggact ggaatgtggt cagagatgac tcagtggttc
15481 tagcctggga gctgagccct ggtgagttct gggatgaggt gacggcctgg cgcaagagca
15541 cgggcaccca gagggtctcc cttgcctttg tctattgtgt catgtgggtt ggagaaaatc
15601 ctggtcaccc ataacccact atccacttag agccctcgag tcttaccgat ttgttcttaa
15661 caatggcatg agcctgttcc tgccttttta gtttaaggcc cgtgagcgtc tttggcagcc
S5 15721 tgtggctctg ggtgggcacc tggcctgcat tcacctgccc cgccctcacc tgccccgccc

15781 tcacctgtcc ccgccccgca gggagctcga ggagcagcac ggcatccact gcaacatgac
15841 gttactcttc tccttcgccc aggctgtggc ctgtgccgag gcgggtgtga ccctcatctc
15901 cccatttgtt gggcgcatcc ttgattggca tgtggcaaac accgacaaga aatcctatga
15961 gcccctggaa gaccctggtg agggtccctc tgtggtaatg gggtaagggg agcagcctca
16021 gcagcacctc aggcaggaag agcccgagac ggagctgccg catcaacaag cagtgaagtg
16081 cacaggtcgg cgcggggcaa gcaggggtgg cggctcaagg taatgcagcc ggcaggcact
16141 gggaaggcag gtggggtggt acctctgccg aaccttcctg gtcacagctt ggtctctttc
16201 caggggtaaa gagtgtcact aaaatctaca actactacaa gaagtttagc tacaaaacca
16261 ttgtcatggg cgcctccttc cgcaacacgg gcgagatcaa agcactggcc ggctgtgact
16321 tcctcaccat ctcacccaag ctcctgggag agctgctgca ggacaacgcc aagctggtgc
16381 ctgtgctctc agccaaggcg ggtgaggccc cactgcccag ctgtggctcc tgctcgggca
A6 16441 aggccagcac tgccgtgcca cgctgccctg tgggtgatcc ctcccacctg tggggcgaac
16501 tcatcttggg aaacatgaag gtgaaataaa actcaacgga aggcttggct ttcctaacac
16561 atctcactca agtcatcaac caaggctctg tgggctgaac ccacttccaa cgtgagcctt
16621 gctggggcca aggcctggcc ctggtgggaa atgcttgggt gcagcagttc aagccctgaa
S7 16681 cccaaggggc caaggtgggc agggacatgg agcaggcatg gaaggctggt tcttgtcccc
16741 cagcccaagc cagtgacctg gaaaaaatcc acctggatga gaagtctttc cgttggttgc
16801 acaacgagga ccagatggct gtggagaagc tctctgacgg gatccgcaag tttgccgctg
16861 atgcagtgaa gctggagcgg atgctgacag tgagtgttgt gtgtgggtac ctacatatgc
A7 16921 caagccctat gagagcccgg gcctgggcgt cggggttcaa gctgggcaga gttaaaactt
16981 tggtgagacc ccaggtctca ttcacgtgct gtccagcaag tggggcctgt tgaggccatc
17041 ccagggtgga gggtgcatgg cacccagggg tgccccc agg atgcttggtg ggtcctggct
17101 cctctctggc ctgtgtggtg actgtggtat tggccaccct gtggccgtgg cccccacaca
17161 gagtgcagac cccacaaggg cctccctcct tccacatggt gccttgtgag gcttcaaggt
S8 17221 gcacaaggta gggtggggag acacagctcg tgctctgttt gtttctagga acgaatgttc
17281 aatgcagaga atggaaagta gcgcatccct gaggctggac tcc*agatct*g caccgccggc
17341 cagctgggat ctgactgcac gtggcttctg atgaatcttg cgttttttac aaattggagc
A8 17401 agggacagat catagatttc tgattttatg taaaatttttg cctaatacat taaagcagtc
17461 acttttcctg tgctgtttc

Figure S2

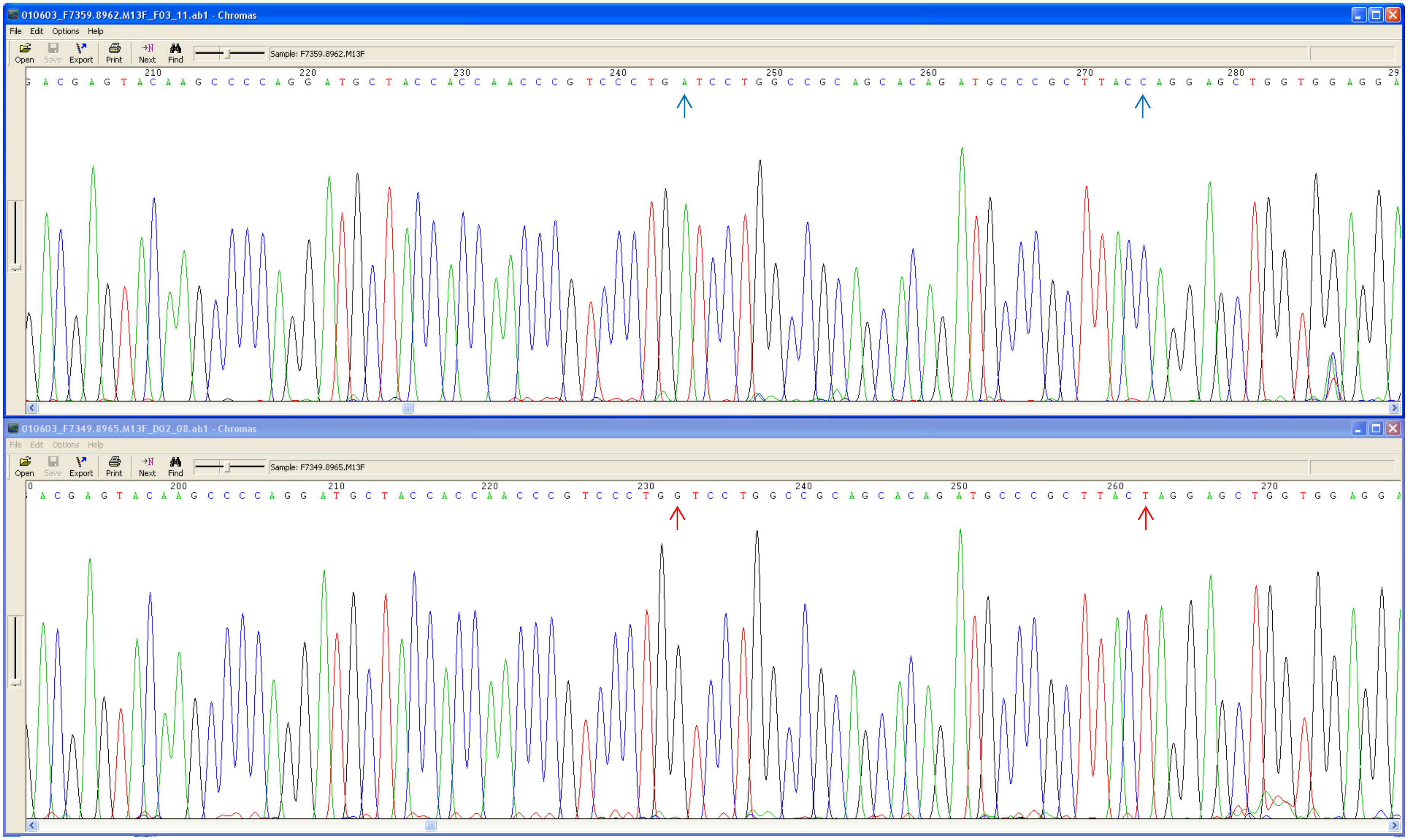
010603_F7359.8962.M13F_F03_11.ab1 - Chromas
Sample: F7359.8962.M13F
010603_F7349.8965.M13F_D02_08.ab1 - Chromas
Sample: F7349.8965.M13F

Figure S3

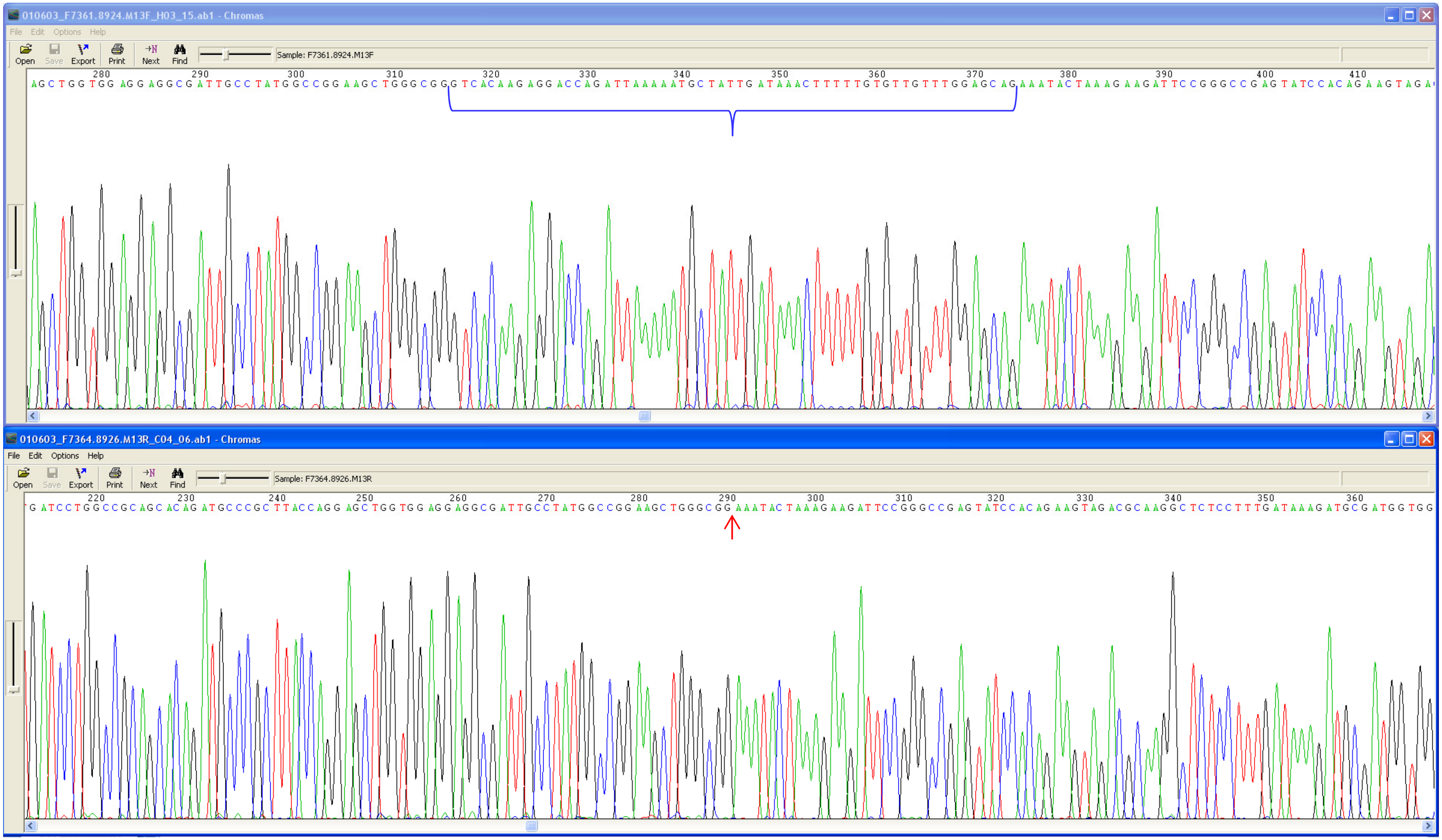

010603_F7361.8924.M13F_H03_15.ab1 - Chromas
Sample: F7361.8924.M13F
010603_F7364.8926.M13R_C04_06.ab1 - Chromas
Sample: F7364.8926.M13R

Figure S4A

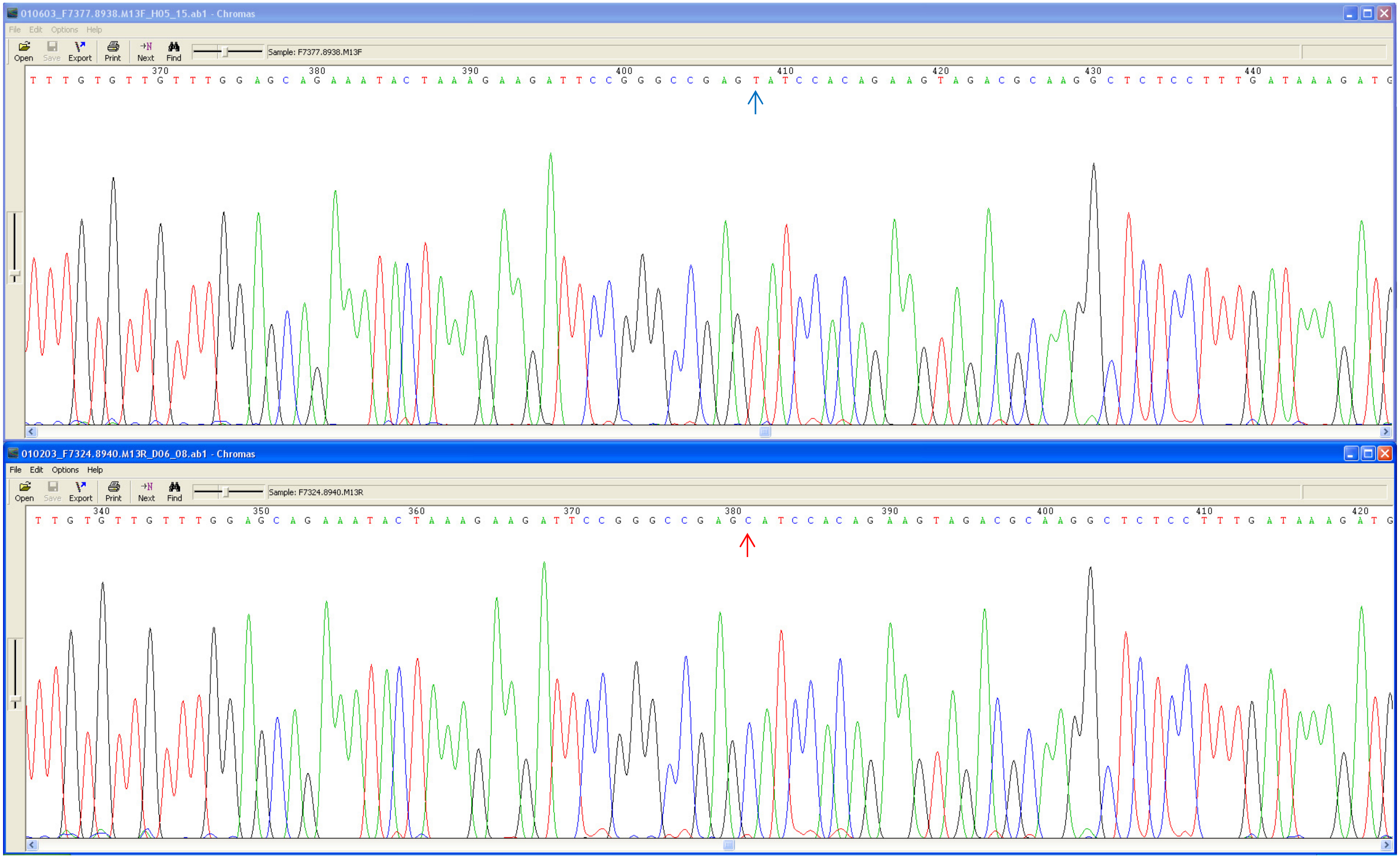
010603_F7377.8938.M13F_H05_15.ab1 - Chromas
File Edit Options Help
Open Save Export Print Next Find
Sample: F7377.8938.M13F
010203_F7324.8940.M13R_D06_08.ab1 - Chromas
File Edit Options Help
Open Save Export Print Next Find
Sample: F7324.8940.M13R

Figure S4B

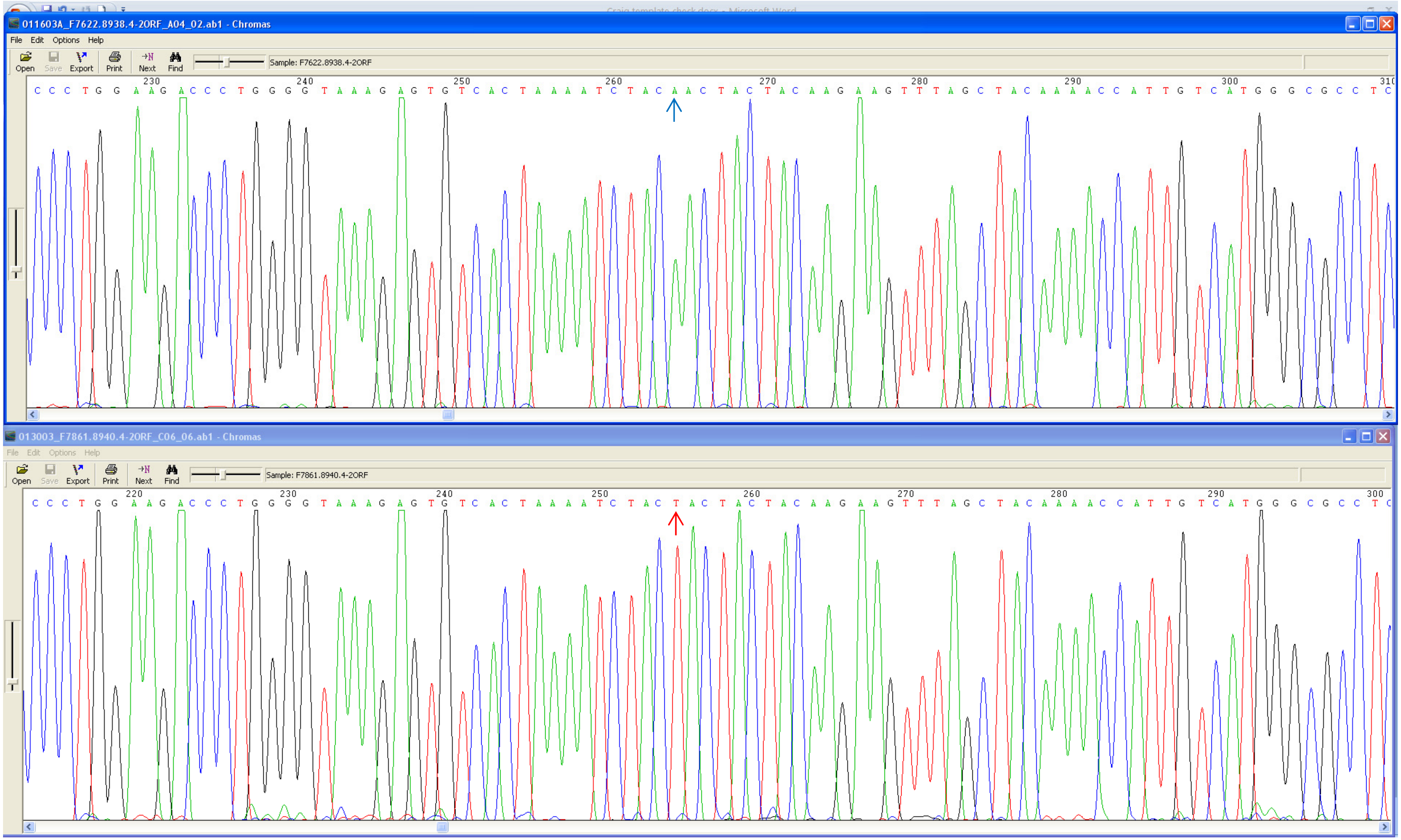
011603A_F7622.8938.4-2ORF_A04_02.ab1 - Chromas
Sample: F7622.8938.4-2ORF
013003_F7861.8940.4-2ORF_C06_06.ab1 - Chromas
Sample: F7861.8940.4-2ORF

Figure S4C

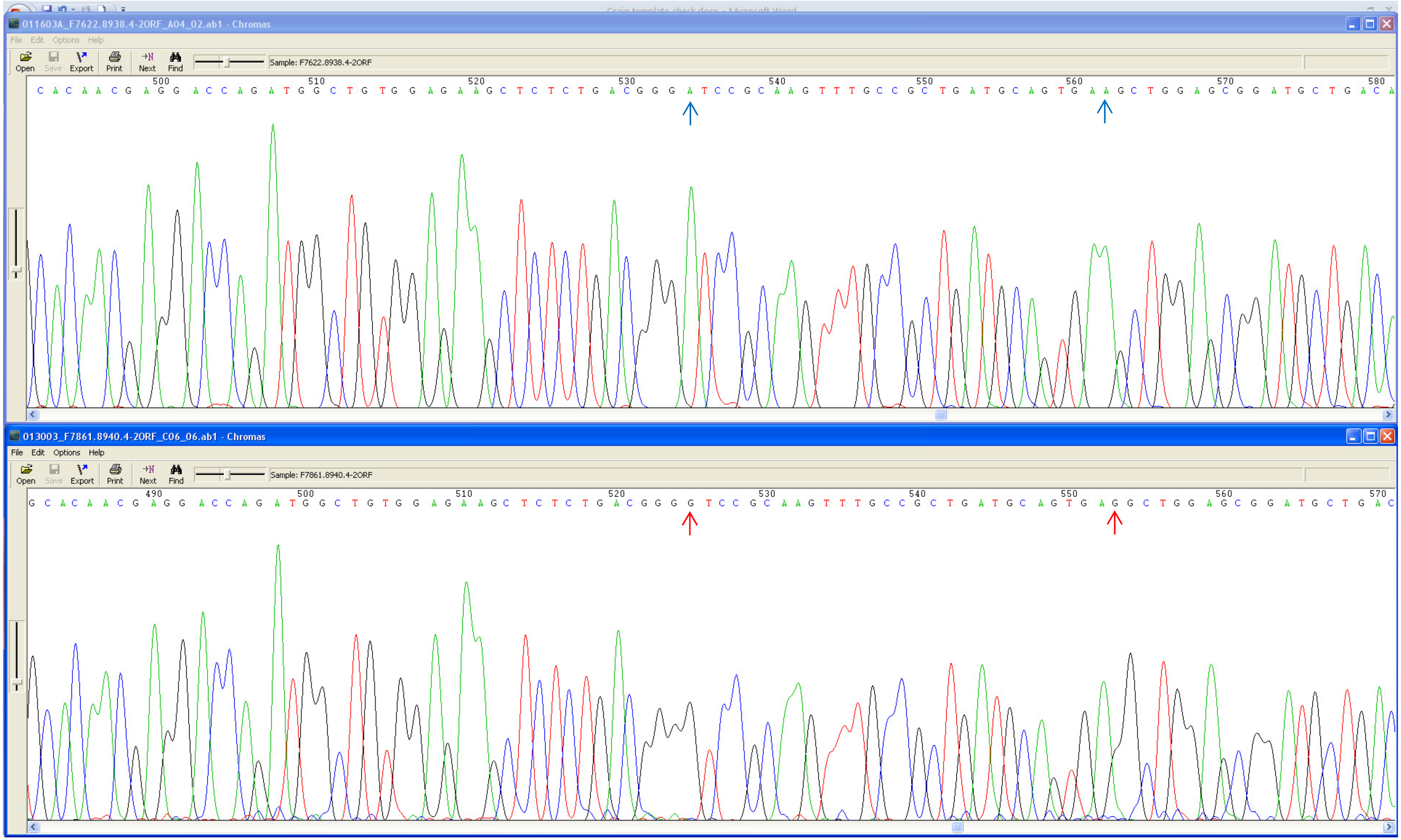

011603A_F7622.8938.4-2ORF_A04_02.ab1 - Chromas
Sample: F7622.8938.4-2ORF
013003_F7861.8940.4-2ORF_C06_06.ab1 - Chromas
Sample: F7861.8940.4-2ORF

Figure S5

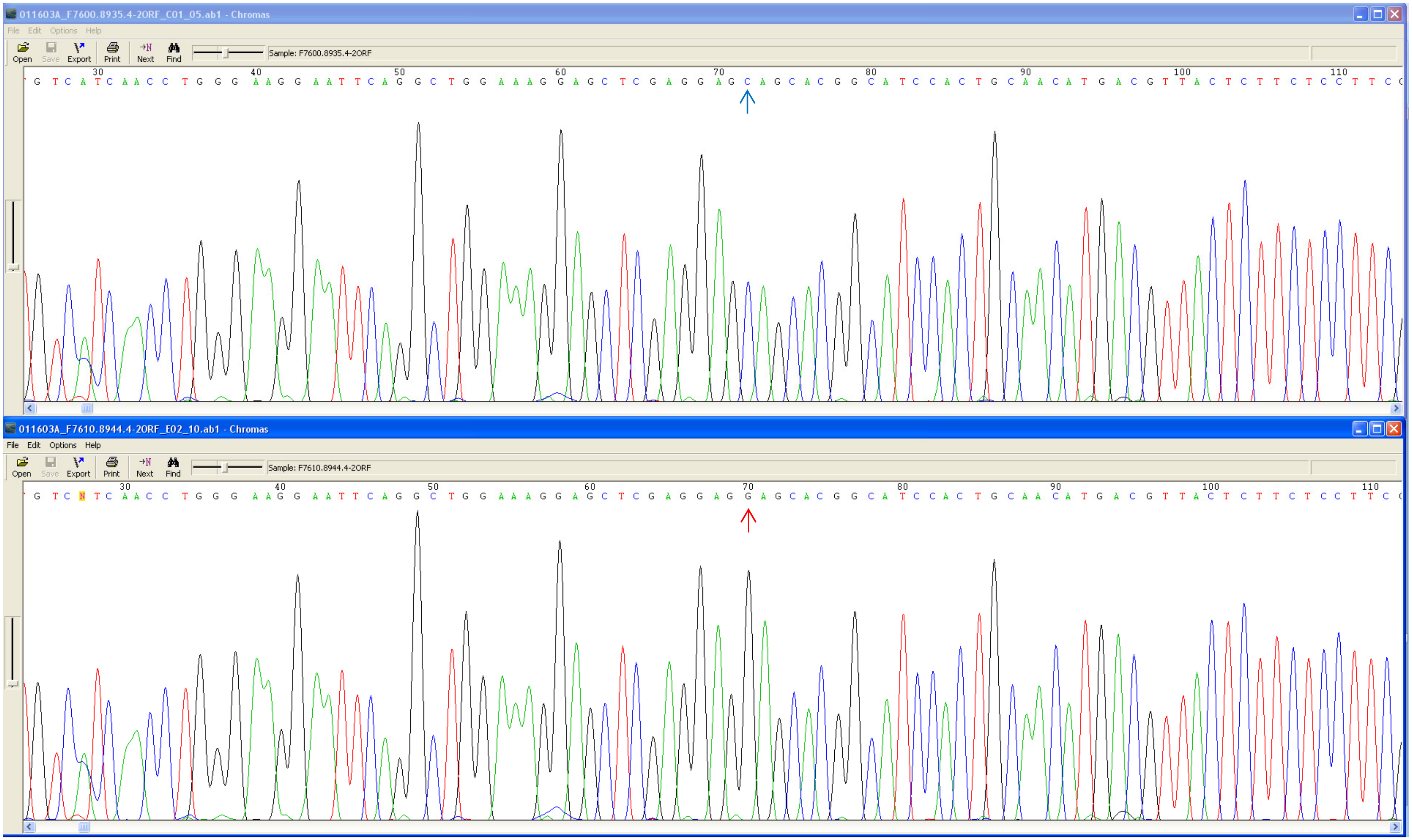
011603A_F7600.8935.4-2ORF_C01_05.ab1 - Chromas
Sample: F7600.8935.4-2ORF
011603A_F7610.8944.4-2ORF_E02_10.ab1 - Chromas
Sample: F7610.8944.4-2ORF

Figure S6A

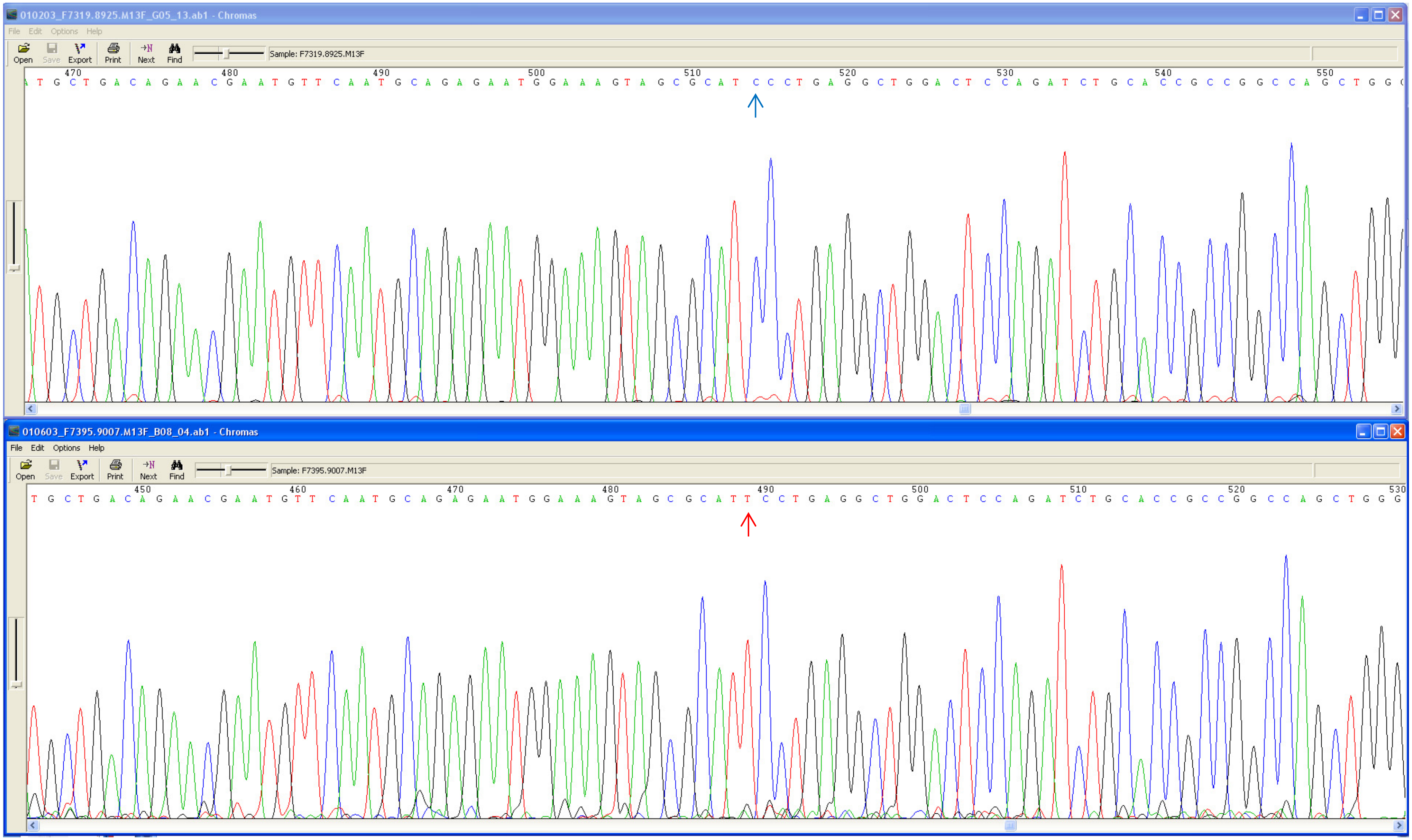

010203_F7319.8925.M13F_G05_13.ab1 - Chromas
File Edit Options Help
Open Save Export Print Next Find
Sample: F7319.8925.M13F
010603_F7395.9007.M13F_B08_04.ab1 - Chromas
File Edit Options Help
Open Save Export Print Next Find
Sample: F7395.9007.M13F

Figure S6B

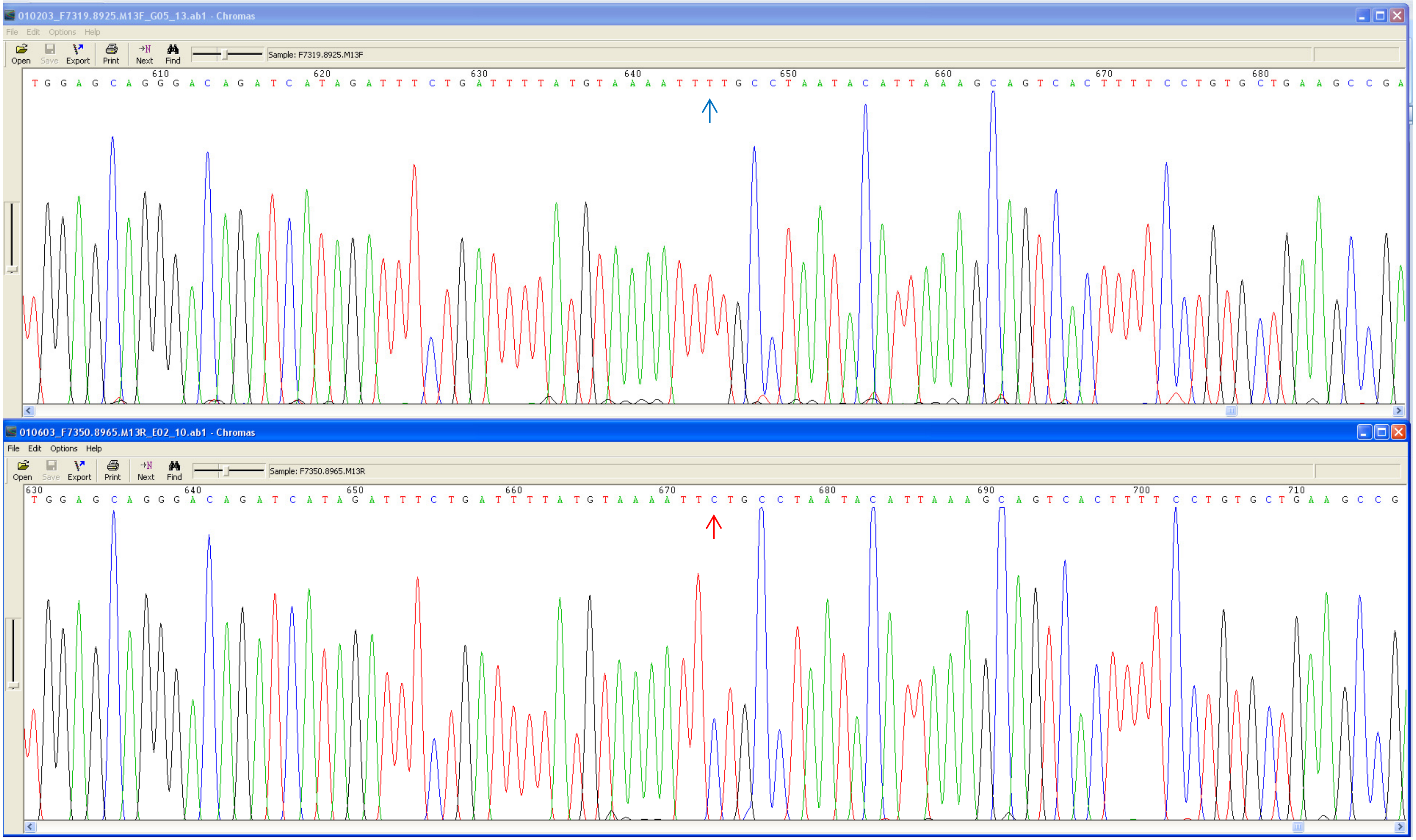
010203_F7319.8925.M13F_G05_13.ab1 - Chromas
File Edit Options Help
Open Save Export Print Next Find
Sample: F7319.8925.M13F
610 620 630 640 650 660 670 680
T G G A G C A G G G A C A G A T C A T A G A T T T C T G A T T T T A T G T A A A A T T T T G C C T A A T A C A T T A A A G C A G T C A C T T T T C C T G T G C T G A A G C C G A
010603_F7350.8965.M13R_E02_10.ab1 - Chromas
File Edit Options Help
Open Save Export Print Next Find
Sample: F7350.8965.M13R
630 640 650 660 670 680 690 700 710
T G G A G C A G G G A C A G A T C A T A G A T T T C T G A T T T T A T G T A A A A T T C T G C C T A A T A C A T T A A A G C A G T C A C T T T T C C T G T G C T G A A G C C G

Figure S8

A

UTSW 38

UTSW 37

B

UTSW 38

UTSW 37

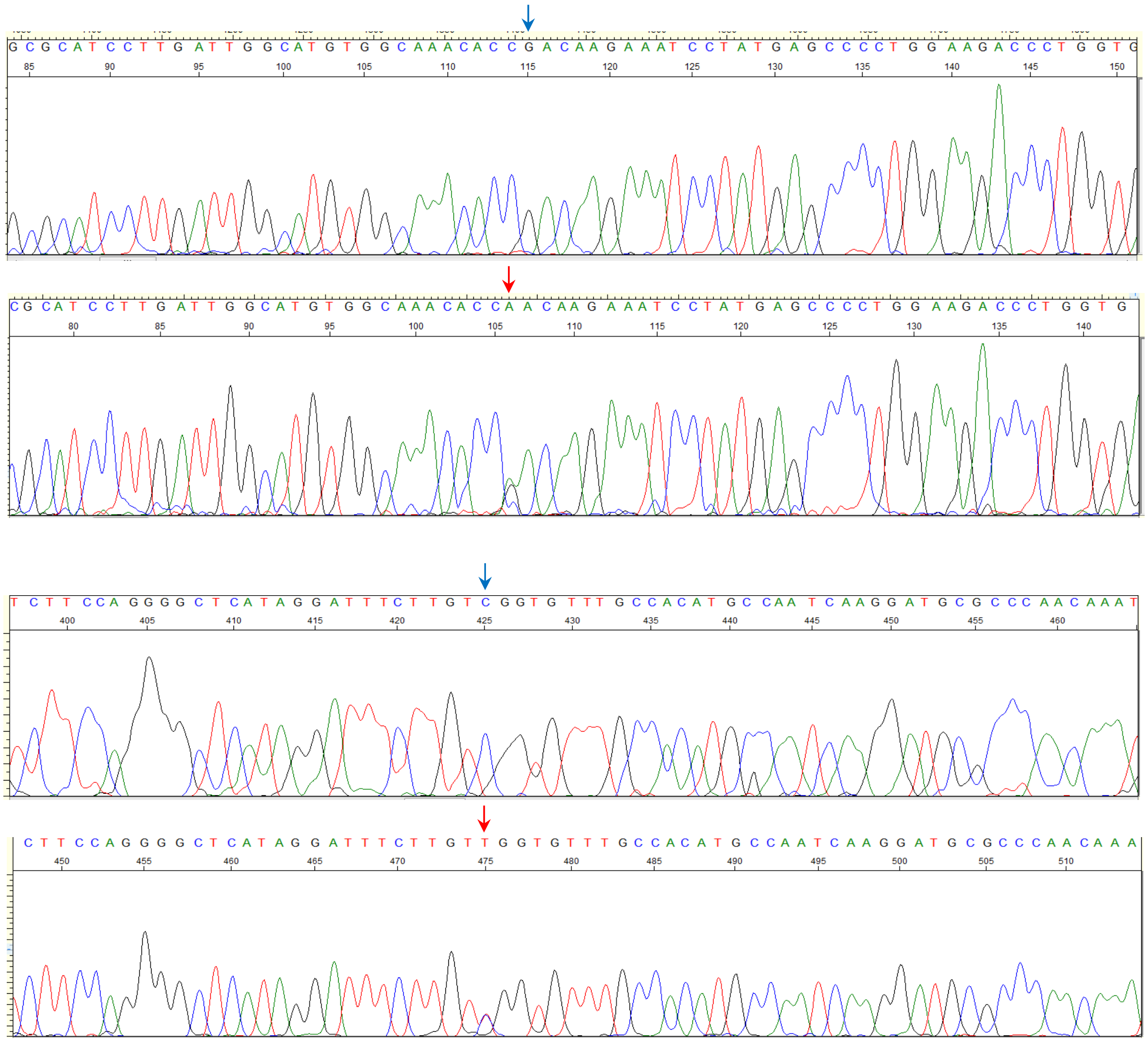

Figure S9

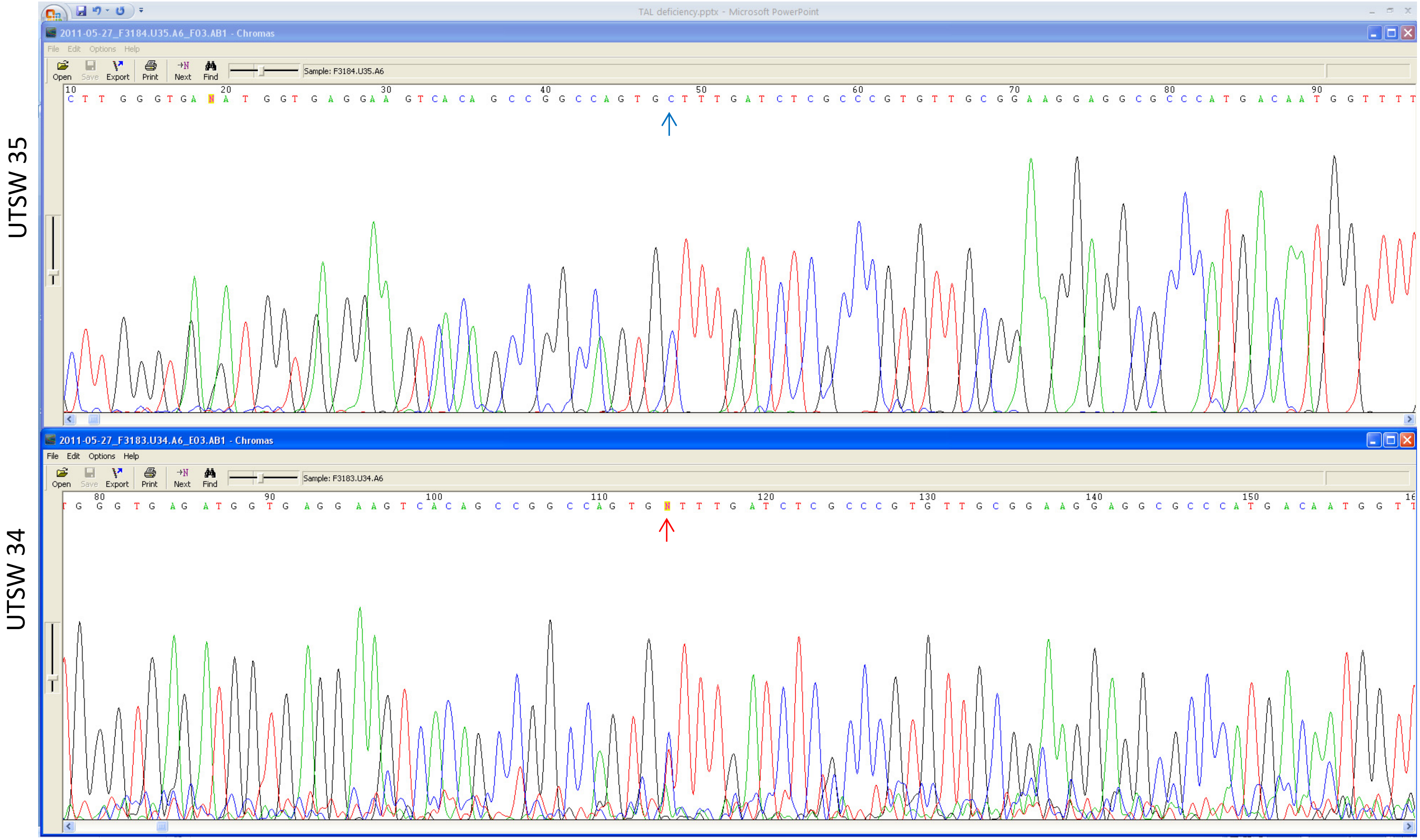
TAL deficiency.pptx - Microsoft PowerPoint
2011-05-27_F3184.U35.A6_F03.AB1 - Chromas
Sample: F3184.U35.A6
UTSW 35
2011-05-27_F3183.U34.A6_E03.AB1 - Chromas
Sample: F3183.U34.A6
UTSW 34

Figure S10

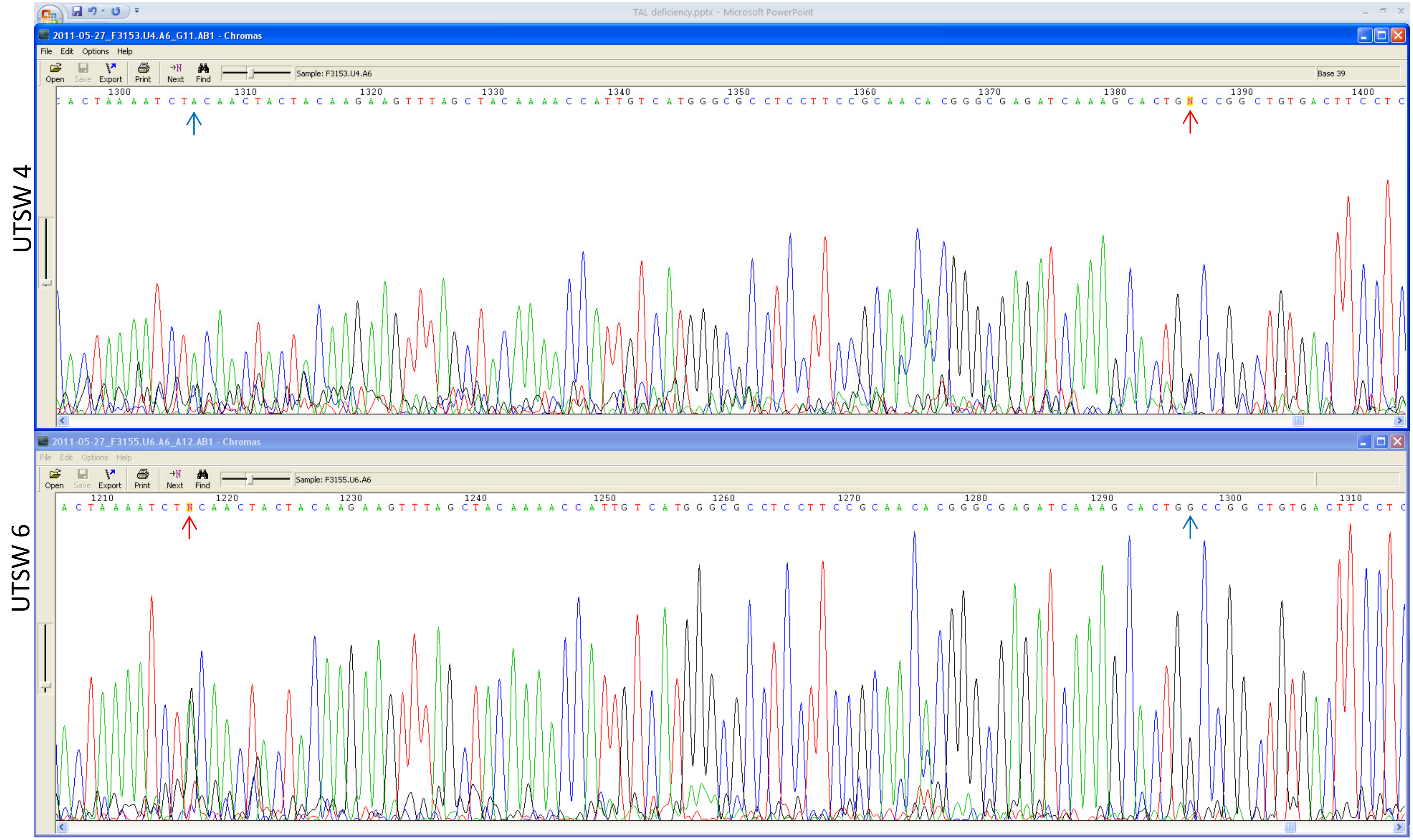

Figure S10

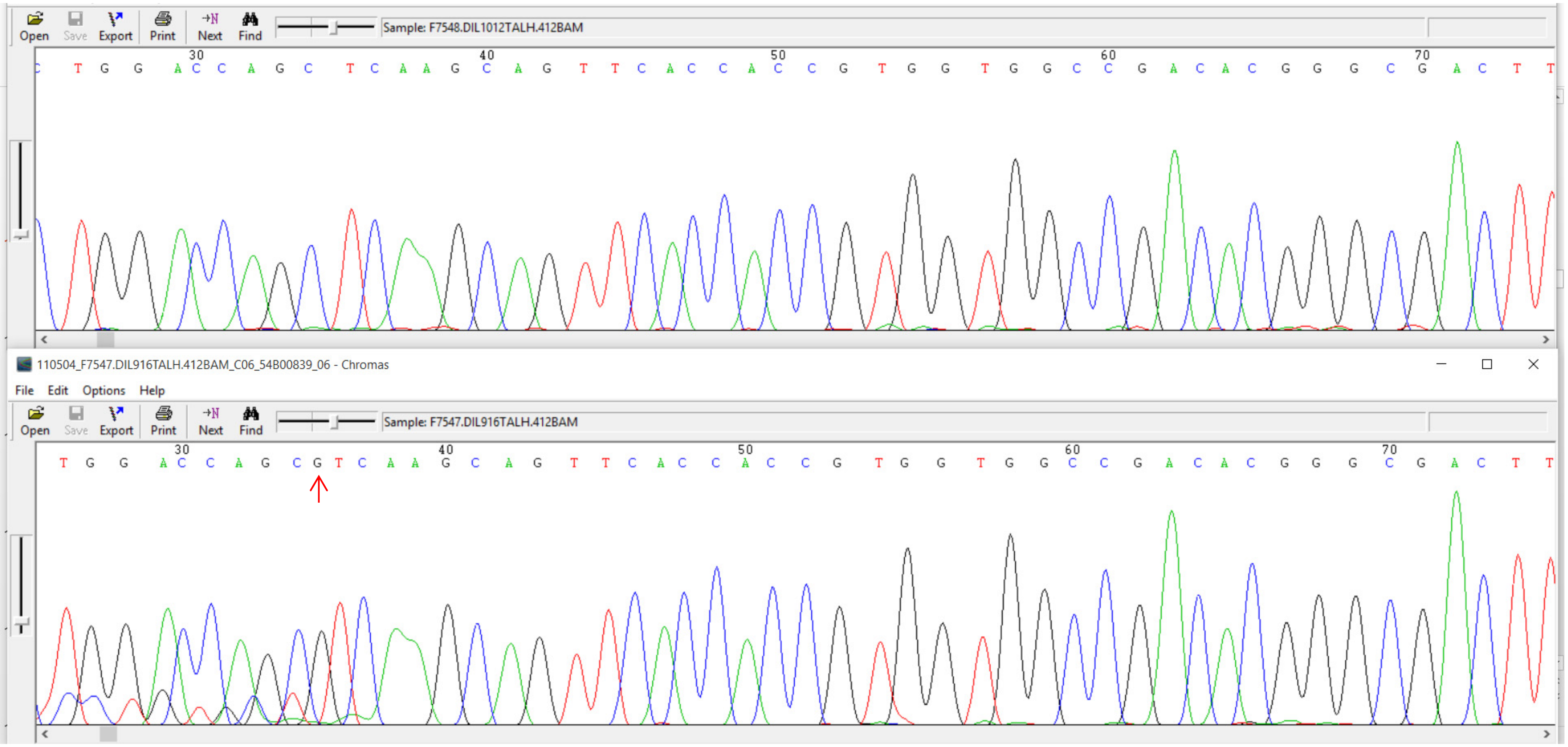
Open
Save
Export
Print
Next
Find
Sample: F7548.DIL1012TALH.412BAM
110504_F7547.DIL916TALH.412BAM_C06_54B00839_06 - Chromas
File
Edit
Options
Help
Open
Save
Export
Print
Next
Find
Sample: F7547.DIL916TALH.412BAM

Figure S11

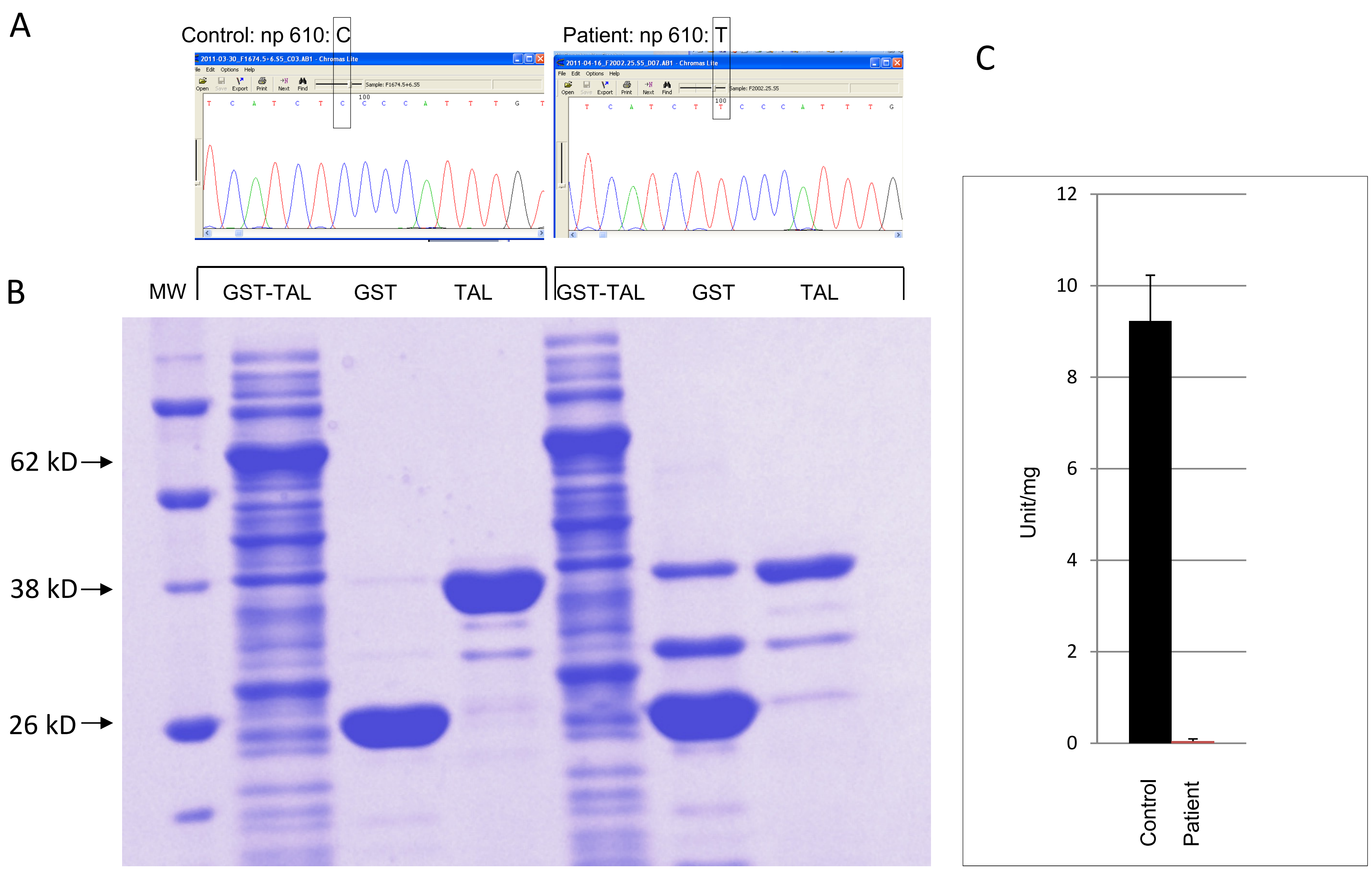
A
Control: np 610: C
Patient: np 610: T
B
MW
GST-TAL
GST
TAL
GST-TAL
GST
TAL
62 kD
38 kD
26 kD
C
12
10
8
6
4
2
0
Unit/mg
Control
Patient
